# A Mineralocorticoid Receptor Deficiency in Myeloid Cells Reduces Liver Steatosis by Impairing Activation of CD8^+^ T Cells in a Nonalcoholic Steatohepatitis Mouse Model

**DOI:** 10.3389/fimmu.2020.563434

**Published:** 2020-12-16

**Authors:** Natalia Muñoz-Durango, Marco Arrese, Alejandra Hernández, Evelyn Jara, Alexis M. Kalergis, Daniel Cabrera

**Affiliations:** ^1^ Millennium Institute on Immunology and Immunotherapy, Departamento de Genética Molecular y Microbiología, Facultad de Ciencias Biológicas, Pontificia Universidad Católica de Chile, Santiago, Chile; ^2^ Departamento de Gastroenterología, Escuela de Medicina, Pontificia Universidad Catolica de Chile, Santiago, Chile; ^3^ Centro de Envejecimiento y Regeneración (CARE), Departamento de Biología Celular y Molecular, Facultad de Ciencias Biológicas, Pontificia Universidad Católica de Chile, Santiago, Chile; ^4^ Departamento de Ciencias Básicas, Facultad de Ciencias, Universidad Santo Tomás, Santiago, Chile; ^5^ Departamento de Endocrinología, Facultad de Medicina, Pontificia Universidad Católica de Chile, Santiago, Chile; ^6^ Facultad de Ciencias Médicas, Universidad Bernardo O Higgins, Santiago, Chile

**Keywords:** non-alcoholic steatohepatitis, steatohepatitis, fatty liver, inflammation, fibrosis, mineralocorticoid receptor, myeloid cells

## Abstract

**Background and Aims:**

The mineralocorticoid receptor (MR) and renin-angiotensin-aldosterone system (RAAS) are implicated in non-alcoholic liver fatty disease (NALFD). However, inflammatory mechanisms linking MR and RAAS with disease pathology remain unclear. Here we aimed to evaluate the contribution of myeloid MR to the inflammatory response in an animal model of non-alcoholic steatohepatitis (NASH), induced with a methionine-choline deficient diet (MCD).

**Methods:**

Mice with a conditional deficiency of MR in myeloid cells (MyMRKO) and their counterpart floxed control mice (FC) were fed for 18 days with MCD or chow diet, respectively. Serum levels of aminotransferases and aldosterone levels were measured and hepatic steatosis, inflammation and fibrosis scored histologically. Hepatic triglyceride content (HTC) and hepatic mRNA levels of pro-inflammatory pro-fibrotic-associated genes were also assessed. Deep flow cytometric analysis was used to dissect the immune response during NASH development.

**Results:**

MyMRKO mice fed with an MCD diet exhibited reduced hepatic inflammation and lower HTC than controls. Absolute number and percentage of liver inflammatory infiltrate cells (except for CD8^+^ T lymphocytes) were similar in both MyMRKO and control mice fed with an MCD diet but expression of the costimulatory molecule CD86 by dendritic cells and the CD25 activation marker in CD8^+^ T cells were significantly reduced in MyMRKO.

**Conclusions:**

Proinflammatory cells are functionally suppressed in the absence of MR. We hypothesized that loss of MR in myeloid cells reduces lipid accumulation in the liver, in part through modulating the adaptive immune response, which is pivotal for the development of steatosis.

## Introduction

Nonalcoholic fatty liver disease (NAFLD) is the most common cause of chronic liver disease worldwide, with an estimated prevalence of 25%–30% in the general population (1, 2). NAFLD is linked to obesity and occurs as a sequential process that starts with lipid accumulation (steatosis), followed by a local inflammatory response and injury, which ultimately fuels progression to fibrosis (3). The presence of necro-inflammatory changes and fibrosis denotes non-alcoholic steatohepatitis (NASH) (4). Individuals with NASH may develop cirrhosis, which confers the risk of end-stage liver disease and hepatocellular carcinoma (HCC) (5). Importantly, the mechanistic aspects linking inflammation with NAFLD/NASH pathogenesis remain unclear, impeding advancements in prevention and specific treatments.

As potential key contributors to this inflammatory processes are hepatic stellate cells (HSC) (6), which regulate fibrosis development due to their capacity to trans-differentiate into myofibroblast-like cells. Although *In vivo* depletion of HSC has been shown to significantly reduce fibrosis (7), leukocyte infiltration was increased, suggesting that these cells can amplify the response to liver injury. Purified and activated HSC can regulate the function of CD8^+^ T lymphocytes, which displayed lower proliferative index and cytotoxic activity (8). This observation suggests an important link between these cell populations and the pathogenesis of liver fibrosis.

A chronic inflammatory milieu, including both innate and adaptive immune responses, is also crucial for NASH development (6, 9). Chronic inflammation involves damage-associated molecular patterns (DAMPs), inflammasome activation, sensitization of the tissue to adverse effects of lipopolysaccharide (LPS) exposure from the microbiome, and lipid peroxidation-derived antigens also can contribute to this pathology (10). Hepatocyte injury or death due to the inflammatory response leads to the release intracellular contents (DAMPs) that further amplify inflammation promoting activation of resident Kupffer Cells (KCs) and recruitment of innate immunity cells such as monocytes and macrophages. On the other hand, dendritic cells (DCs) play an unclear role during NASH/NAFLD. While these cells may be immunoregulatory, particularly for CD8^+^ T lymphocytes, an absence of some DCs is associated with a worsened steatohepatitis phenotype (11). Neutrophils are considered a hallmark for NASH in mice and humans (9). Thus, the neutrophil: lymphocyte ratio has been proposed as a good marker to predict steatohepatitis and fibrosis in patients with NAFLD (12). Furthermore, a crosstalk between neutrophils and HSC is thought to be required for maintaining the oxidative/proinflammatory loop that promotes liver fibrosis (13).

The contribution of adaptive immunity to NASH development is underscored by the observation that T cell-deficient animals are resistant to this ailment (14). Livers of mice fed a methionine-choline deficient (MCD) diet display significantly increased numbers of CD3^+^ T cells with an IFN-γ-secreting Th1 phenotype, as described in humans (15). However, the contribution of these finding to liver disease remains unclear. Further, animals fed an MCD diet exhibit an increased liver infiltration of CD4^+^ and CD8^+^ T cells, as well as antibodies against malonyldialdehyde (16), which is suggestive of a role for adaptive immunity during NASH (16). *In vitro* studies of the Th17 immune response in HepG2 cells have shown that IL-17 potentiates steatosis in the presence of free fatty acids, while human liver histopathology analyses have identified a significant infiltration of IL-17^+^ cells (17). Finally, CD8+ derived IFN-γ has a direct implication in steatosis development (18).

Liver cells such as hepatocytes, HSC, endothelial cells, and KCs express receptors for angiotensin and mineralocorticoid hormones (MR), which are involved in the Renin-Angiotensin-Aldosterone system (RAAS). RAAS is implicated in inflammatory processes that lead to fibrosis in NASH (19). Binding of angiotensin or mineralocorticoid hormones to their respective receptors can promote oxidative stress, inflammation, and fibrosis (20). RAAS-related molecules are often produced under pathological conditions, prompting research for the identification of antagonists and blockers of RAAS as pharmacological treatments (21).

Previously we reported that DCs express MR and respond to aldosterone stimulation, polarizing CD4^+^ T lymphocytes toward a Th17 phenotype and inducing activation of CD8^+^ T lymphocytes (22, 23). Similarly, in a rat hypertension model, the altered balance between CD4^+^ Th17 and T regulatory cells was restored after MR antagonism (24, 25). Further, an inflammatory phenotype induced by aldosterone in peritoneal macrophages resembled a “*classical*” or M1 activation (26). This phenotype was not observed after pretreatment eplerenone, a MR antagonist (26). In a mouse model with conditional myeloid-cell knockout of MR (MyMRKO), peritoneal macrophages displayed an “alternative” or M2 activation (26).

Given the contribution of immunity and inflammation to the pathology of NASH, and the potential for MR to modulate immune responses, MR antagonism or MR knock-down have been evaluated to prevent steatosis and fibrosis in NASH dietary animal models using either high fat (27) or choline-deficient-amino-acid-defined (CDAA) diets (28). MyMRKO mice fed with high-fat diet demonstrated that the MR expressed in myeloid cells mediates the cellular crosstalk between KCs and hepatocytes and that the loss of myeloid MR prevents steatosis (29). While these studies reveal important links between MR and immune responses that may be involved in NASH pathogenesis, further characterization of inflammatory cells changes related with steatosis development is lacking. Here, we assessed inflammatory and liver histopathologic changes in MyMRKO mice fed with MCD diet to induce NASH.

## Materials and Methods

### Ethics Statements

All mice were maintained under pathogen-free conditions in the facilities of Pontificia Universidad Católica de Chile (Santiago, Chile) at 25°C and 12 h:12 h light:dark cycles and consuming food and water *ad libitum*. The protocols were conducted in agreement with the National Research Council (NRC) publication Guide for Care and Use of Laboratory Animals, 8th edition (2011, US National Academy of Sciences). Animal protocols were approved by the Ethics Committee for Animal Welfare from the School of Medicine of the Pontificia Universidad Católica de Chile (CEBA #170525006) and supervised by an institutional veterinarian.

### Mouse Breeding and Genotyping

C57BL/6 wild-type mice were obtained from The Jackson Laboratory (Bar Harbor, ME) and were used at 6–8 weeks of age. OVA-specific OT-I and OT-II transgenic mice expressing specific TCRs for H-2Kb/OVA257–264 and I-Ab/OVA323–339 respectively, were obtained from Dr. R. Steinman (The Rockefeller University, New York, NY).

To perform the experiments, we used MR knockout conditional mice in myeloid cells (MyMRKO) which was kindly, donated by donated by Dr. Richard M. Mortensen (University of Michigan, Michigan, USA) and from MTA with Dr. Günther Schütz (DKFZ, Im Neuenheimer Feld, Heidelberg, Germany) (26). These conditional knockout mice were obtained by crossing floxed MR*^flox/flox^* mice with floxed MR*^flox/flox^*/Cre-recombinase mice, which express this enzyme under LysM promoter (LysMCre) (26). Genotyping of this animals is described in the supplementary methods section. For all the experiments we used MR*^flox/flox^* as control mice (FC). In addition, we evaluate the efficiency of this conditional Knock out by evaluating the expression of MR in blood-derived myeloid cells (**Supplementary Figure 1A**).

### Non-Alcoholic Steatohepatitis Induction With MCD Diet

After genetic screening, 10-week-old male and female mice were used to induce NASH using methionine-choline deficient diet (MCD; Dyets Inc. Bethlehem, PA, USA). Deficiency in both nutrients affects very low-density lipoprotein (VLDL) assembly and impairs lipid exportation from liver to peripheral tissues, giving the phenotype of macrovesicular liver steatosis (30). Control groups were fed with conventional chow diet (Lab Diet Prolab rmh 3000, USA). MCD diet is commonly used to evaluate, in short periods of time, histological features associated with NASH, including fibrosis (31). Animals were divided in 4 experimental groups each with n = 4-8 animals and the complete experiment was performed twice. **1)** Control group: floxed mice (FC) fed chow control diet; **2)** MCD FC group: FC mice fed MCD diet to induce NASH; **3)** Control MyMRKO group: MyMRKO mice fed chow control diet; and **4)** MCD MyMRKO group: MyMRKO mice fed MCD diet to induce NASH. Pellets and water were available *ad libitum* for consumption, and MCD and chow diet pellets were changed every other day. Animals were observed daily to verify health status and were weighted twice per week as an indicator of disease outcome. Humanitarian endpoints were applied if mice displayed any signs of suffering according to protocol number 61005001 approved by Pontificia Universidad Católica de Chile.

### Histology Analyses and NAS Score

Histological analyses were performed in paraformaldehyde-fixed liver sections obtained from liver right lobes as previously described. Sections were stained with Hematoxylin/Eosin to evaluate liver tissue architecture (lipid droplets, cell infiltration, cell injury, etc.). Liver steatosis was specifically assessed by Oil Red O staining in frozen liver cryosections (Abcam, USA). Liver fibrosis was assessed using Sirius Red, whereby liver sections were incubated for 2 h at room temperature with an aqueous solution of saturated picric acid containing 0.1% Fast Green FCF and 0.1% Direct Red. Red-stained collagen fibers were quantitated by digital image analysis (ImageJ, NIH, US). Steatosis, inflammation, and ballooning were graded on the basis of the NAFLD activity score (NAS) criteria by an experienced pathologist. NAS includes the punctuation of steatosis, inflammation, ballooning, and fibrosis in routinely stained liver sections (32). All images were obtained using the Aperio AT2 slide scanner and analyzed with the Aperio ImageScope - Pathology Slide Viewing Software and ImageJ software (ImageJ, NIH, US).

### Tissue Digestion to Perform Flow Cytometric Analyses

To characterize the leukocyte infiltration in livers we followed with modifications the process described by VanSaun et al. (33). Briefly, livers and spleens were collected from each animal after cardiac perfusion with 10 ml of heparinized-Krebs Ringer Buffer (KRB) made in house. Then, livers were weighed and divided in three different sections, two of them to perform histological analysis and the third to achieve cytometric analysis. The final section was cut in small pieces and resuspended in KRB containing Collagenase IV (500 U/ml, Life Technologies 17104019), DNAseI (1500 U/ml, Merck 11284932001), CaCl_2_ (2mM, Winkler 10035-04-8) and MgCl_2_ (2mM, Winkler 7791-18-6), and incubated for 30 min at 37°C in warm bath, with constant agitation. Samples were transferred to cell strainers (70-µM, BD Falcon) and were mechanically disrupted, washed twice with PBS containing 2 mM EDTA (Merck ED-100G) and 0.5% BSA (Merck A9418) at 500 x g for 5 min at room temperature. Cell suspensions were centrifuged at 30 x g for 6 min to pellet hepatocytes and collect the leukocytes in supernatant. This cell suspension was centrifuged at 300 x g during 10 min to pellet leukocytes to be processed to flow cytometric analysis described in supplementary methods.

### Statistical Analyses

Data are presented as mean ± standard error of mean (SEM). Some analyses were performed by one-way ANOVA followed by Tukey post-test. When only two groups were compared, analysis were done by unpaired t test with Welch´s correction. Correlation analysis was performed first by a linear regression, followed by Spearman analysis of correlation. In all cases a p value < 0.05 was considered statistically significant.

## Results

### MR Deletion Attenuates Liver Steatosis and inflammation in Mice Fed With MCD Diet

First, all experiments were performed with conditional knockout mice in which the expression of MR was specifically abolished in myeloid cells (26). Myeloid MR knockout mice (MyMRKO) mice on the C57BL/6J (WT) background were obtained by crossing homozygous floxed MR mice (MR*^flox/flox^*, abbreviated in this manuscript as FC) with homozygous floxed MR mice containing LysM-Cre (MR*^flox/flox^*;LysM-Cre×MR*^flox/flox^*). FC were used as control mice in all experiments. MR deletion-efficiency in blood-derived myeloid cells obtained from WT, FC, and MyMRKO mice was evaluated by qPCR. As shown in **Supplementary Figure 1A**, a significant reduction in the expression of MR (82,7%) was observed in MyMRKO mice, as compared to WT or FC controls. Then, to promote NASH development, MyMRKO mice and their counterpart FC mice were fed for 18 days with MCD or chow diet, respectively. Bodyweight was measured twice weekly to assess health status. Both FC and MyMRKO mice fed with the MCD diet exhibited progressive bodyweight reductions with no differences between groups (**Supplementary Figure 1B**). In addition, both MCD-fed groups of mice had higher serum aminotransferases (Alanine transaminase and Aspartate transaminase) compared to control mice fed with a chow diet (**Supplementary Figures 1C, D**), indicating liver damage.

**Figure 1:**
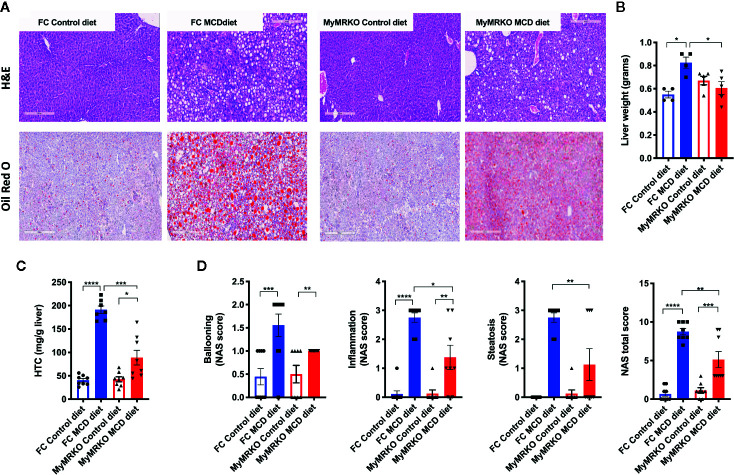
Livers from myleoid mineralocorticoide receptor knockout (MyMRKO) mice present lower lipid content than FC Control mice. Histological changes and NAS score were evaluated by H&E and Oil Red O staining **(A)**. Representative liver micrograph from each experimental group (bars in gray represents a scale of 300 μm) **(B)**. Liver weight in grams **(C)**. Hepatic triglyceride content (HTC) **(D)**. NAFLD activity score (NAS) total scores and steatosis criterion scores. Statistical analysis was performed with one-way ANOVA comparing all treatments, with Tukey post-test. Only the steatosis criteria **(D)** were analyzed by unpaired t-test with Welch´s correction because both groups with a control diet only presented zero values. Each scoring was performed in blinded fashion from an n= 4–5 animals per group. All figures display mean ± SEM. Statistical differences were considered significant according to *p < 0.05 **p < 0.01 ****p < 0.001 ****p < 0.0001.

Next, livers were histologically analyzed by hematoxylin and eosin (H&E) and Oil Red O staining (**Figure 1A**, upper and bottom panel, respectively). Livers from MyMRKO and FC mice fed with a chow diet displayed healthy liver architecture, with similar liver weights (in grams) and similar basal concentrations of hepatic triglycerides (**Figures 1B, C**, respectively). According to NAS scoring criteria, neither livers from MyMRKO nor from FC mice fed with chow diet presented signs of ballooning, inflammation, or steatosis (**Figure 1D**), indicating that all control mice (Control diet-fed FC and MyMRKO mice) displayed typical liver structure. In contrast, both MyMRKO and FC mice fed with the MCD diet displayed signs of altered liver structure, such as ballooning, infiltration of immune cells, and higher lipid accumulation than control diet-fed mice (**Figure 1A**). FC mice showed significantly higher liver weight and lipid content than the chow diet-fed group (**Figure 1B**). Further, NAS scoring criteria were statistically significantly higher in FC mice fed with MCD diet than FC mice fed with a chow diet (**Figure 1D**). Although MyMRKO mice fed with the MCD diet also displayed histological alteration, liver weight, and lipid content were not statistically different from the control group (**Figures 1B, C**). Further, MyMRKO mice fed with the MCD diet displayed significantly lower NAS total score than their counterpart FC (**Figure 1D**, right panel). Specifically, MyMRKO showed lower Hepatic triglyceride content (HTC), steatosis, and inflammation score than did FC mice (**Figures 1C, D** (middle panels).

### MR Deletion Does Not Impact Fibrosis Phenotype and Inflammatory Markers

**Figure 2:**
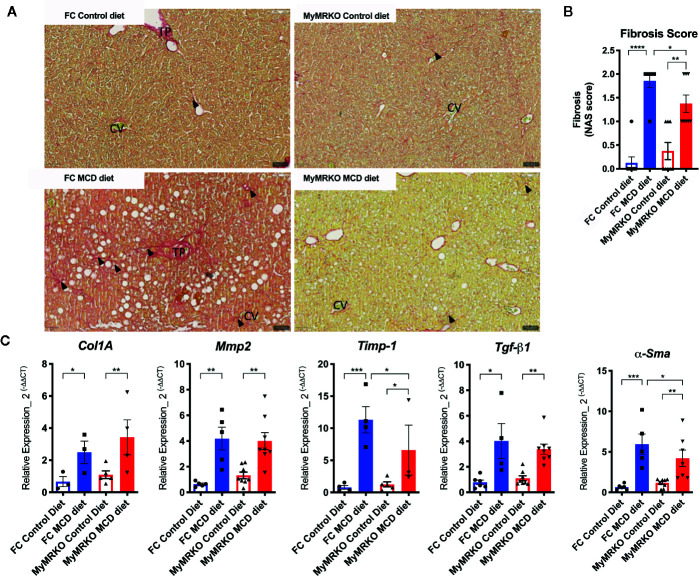
Floxed control (FC) and myleoid mineralocorticoide receptor knockout (MyMRKO) fed with methionie and choline deficient diet (MCD) diet display liver fibrosis and increased expression of fibrosis markers. Additional criteria of the NAFLD activity score (NAS) score (**Figure 1**) are the fibrosis signs, as shown in **(A)**. In this line, the hepatic stellate cells (HSC) are a key modulator of hepatic fibrosis; as shown in **(B)** the expression of *αSma*, a marker of HSC, increased in both groups of mice fed with MCD diet **(C)**. Expression of *Col1A*, *Mmp2*, and *Timp-1*, which are involved in hepatic fibrosis. To normalize the *αSma*, *Col1A*, *Mmp2*, *Timp-1*, and *Tgf-β1* expression, we used *18S* as a housekeeping gene. Each quantification was performed in duplicate from an n=4-8 animals per group. Statistical analysis was performed with one-way ANOVA comparing all treatments, with Tukey post-test. All figures display mean ± SEM. Statistical differences were considered significant according to *p < 0.05 **p < 0.01 ***p < 0.001 ****p < 0.0001.

Fibrosis is one of the hallmarks of NASH disease evolution and is mainly due to the activation of HSCs. Both MyMRKO and FC mice fed with the MCD diet displayed histological signs of fibrosis compared with their chow-fed controls (**Figure 2A**). However, induction of fibrosis was less evident in the MyMRKO MCD fed mice. **Figure 2A** shows through Sirius Red staining that collagen deposition was reduced in the MyMRKO MCD-fed mice compared to FC MCD-fed mice. This result was corroborated by blinded histopathology analyses, showing a reduced histopathological fibrosis score (**Figure 2B**). Besides, these results were supported by the reduction of *αSma* and *Timp-1* expression, which are specific markers of HSC activation to promote fibrosis (**Figure 2C**). However, the expression of *Col1A*, *Mmp2*, and *Tgf-β1* was not affected in the MyMRKO MCD-fed mice as compared to FC MCD-fed mice (**Figure 2C**).

**Figure 3:**
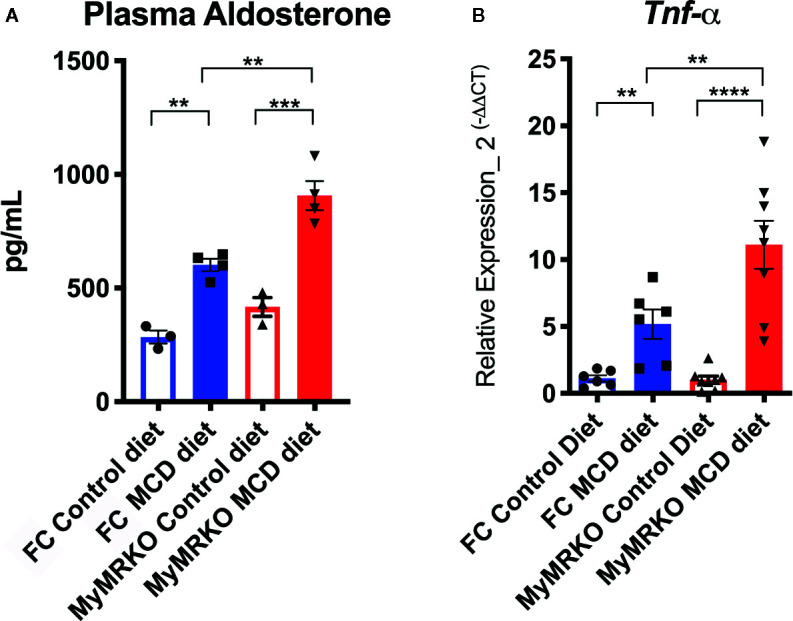
Floxed control (FC) and myleoid mineralocorticoide receptor knockout (MyMRKO) fed with methionie and choline deficient diet (MCD) diet have increased plasma levels of aldosterone and related inflammatory genes. At the experimental endpoint, aldosterone plasma levels were quantified in all experimental groups, as shown in **(A)**. Then, we measured whether genes associated with high levels of aldosterone were positively modulated **(B)**. *Tnf-α* expression was quantified by RT-PCR. 18S was used as a housekeeping gene. To quantify aldosterone, it was necessary to pool plasma from two mice per group to achieve the required volume for quantification. For that reason, graphs display n=4 pooled plasma. RT-PCR was performed in duplicate from an n=7–8 animals per group. Statistical analysis was performed with one-way ANOVA comparing all treatments, with Tukey post-test. All figures display mean ± SEM. Statistical differences were considered significant according to **p < 0.01 ***p < 0.001 ****p < 0.001 ****p < 0.0001.

Overexpression of Timp-1 is associated with high aldosterone levels in primary aldosteronism patients, who frequently develop fibrosis in the heart and kidneys (34). Therefore, we measured aldosterone plasma levels. FC and MyMRKO mice fed with the MCD diet had significantly increased plasma aldosterone levels compared to controls (**Figure 3A**). However, MyMRKO mice showed significantly higher levels than FC mice fed with MCD. This result provides additional support for the role of aldosterone and myeloid MR receptor in NASH’s pathogenesis. After measuring the expression of genes linked to inflammation and fibrosis as well as modulated by aldosterone (26, 35), it was found that *Tnf-α* is highly expressed in MCD-fed mice as compared to chow-fed mice, and it was significantly increased in livers from MyMRKO mice fed with MCD diet compared to chow-fed FC mice (**Figure 3B**).

Expression of cytokines, such *Ifn-γ, Il-1β*, and *Il-18* was measured as markers of T cell response, and innate immunity. These cytokines are detected at high levels in NAFLD patients (36). As expected, both MCD-fed mice displayed higher abundance of mRNA of these inflammatory genes as compared to FC Control diet fed mice (**Supplementary Figure 2**). However, these results showed a paradoxical response in which *Ifn-γ* abundance was reduced while the expression of *Il-1β was increased* in liver samples from MyMRKO MCD-fed mice as compared to FC MCD fed mice.

### Reduced Lipid Accumulation in MyMRKO Mice Associates With Diminished CD8^+^ T Cell Infiltration

**Figure 4:**
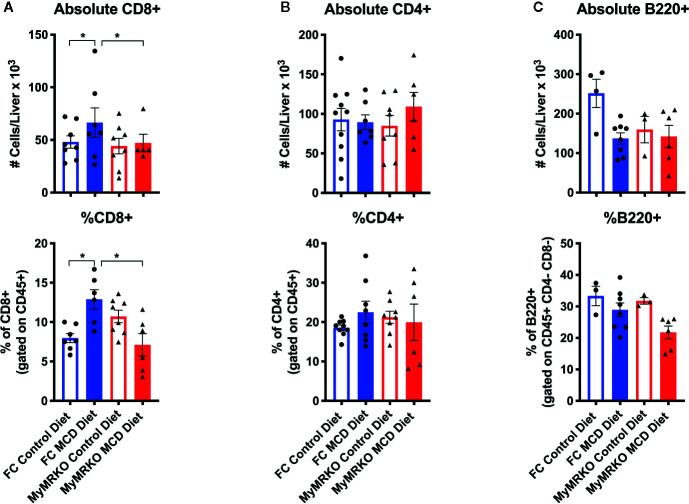
Changes in lymphoid cell infiltration in floxed control (FC) mice is mainly due to CD8^+^ T cells. To measure what type of lymphocytic response is involved in NASH, we evaluated the absolute number and percentage of CD8^+^ T cells **(A)**, CD4^+^ T cells **(B)**, and B220^+^ B cells **(C)** at the endpoint. Flow cytometry characterization was performed individually for each animal per group, achieving n= 7–8, and following the gating strategy described in **Supplementary Figure 1**. Absolute counting was performed with Count Bright™ absolute counting Beads (described in materials and methods). Statistical analysis was performed with one-way ANOVA comparing all treatments, with Tukey post-test. All figures display mean ± SEM. Statistical differences were considered significant according to *p <0 .05.

Because the liver is also recognized as an immunological organ, we sought to dissect the immune response during NASH development, using deep flow cytometric analyses (**Supplementary Figure 3A**). Both groups of mice fed with the MCD diet showed a tendency to increased percentages and absolute numbers of infiltrating hepatic leukocytes or CD45+ cells, as compared to controls (**Supplementary Figure 3B**). For a more specific characterization of these infiltrating leukocytes, we determined T cell markers CD45+, CD3+, CD4+, and/or CD8+, and B cell markers CD45+, CD3-, B220+ (**Supplementary Figure 3A**). From lymphocytic lineage, only CD8+ T cells significantly infiltrated the liver in response to the MCD diet in FC mice (**Figures 4A–C**). CD8+ T cells have been implicated in lipid accumulation in NASH, both in human and animal models (37, 38). Interestingly, while MCD-fed MyMRKO mice showed reduced steatosis (**Figure 1**) and lower expression of hepatic *Ifn-γ* (**Supplementary Figure 2**) as compared to MDC-fed FC mice, these animals displayed reduced CD8^+^ T cell liver infiltration as compared to MCD-fed FC mice (**Figure 4A**, upper and bottom panel, respectively).

Next, we performed correlation analyses with the percentage and the absolute number of CD8+ T cells infiltrating the liver (**Supplementary Figure 4**) and the levels of hepatic triglyceride. As expected, MyMRKO and FC mice fed with a chow diet did not display a significant correlation between CD8+ T cell infiltration and hepatic triglyceride concentrations. Similarly, MyMRKO and FC mice fed with MCD did not display a significant correlation between CD8+ T cell infiltration and lipid accumulation; however, FC mice showed a slight tendency to increase CD8+ T cells infiltration at higher hepatic triglyceride concentrations in MCD-fed mice.

**Figure 5:**
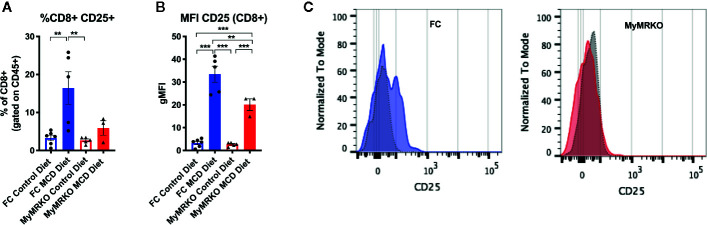
Activation marker CD25 is highly expressed in CD8^+^ lymphocytes from floxed control (FC) mice. Flow cytometry was used to measure the percentage of CD8^+^ lymphocytes that expressed CD25 marker **(A)** and the global expression of CD25 in CD8^+^ lymphocytes or the gMFI **(B)**. A representative image of CD25 expression in myleoid mineralocorticoide receptor knockout (MyMRKO) and FC fed with chow diet (gray dotted histogram) or methionie and choline deficient diet (MCD) diet (colored histogram) is shown **(C)**. Statistical analysis was performed with one-way ANOVA comparing all treatments, with Tukey post-test. All figures display mean ± SEM. Statistical differences were considered significant according to **p < 0.01 ***p < 0.001.

Finally, the percentage of infiltrating CD8^+^ T cells that expressed activation marker CD25 was significantly increased in FC mice fed with MCD than controls, while MyMRKO mice fed with MCD displayed only a mild increase (**Figure 5A**). Additionally, the geometric Median Fluorescence Intensity (gMFI) of this marker was significantly higher in FC mice fed with MCD as compared to both MyMRKO and chow-fed controls (**Figures 5B, C**).

### Liver Infiltrating Myeloid Cells in MyMRKO Mice Fed With MCD Diet Displayed Reduced Expression of CD86 Costimulatory Molecule

Myeloid cells were analyzed and pooled according to their surface marker expression as monocytes, macrophages and KCs, neutrophils, and DCs, as described in the Materials and Methods section. While Monocytes, macrophages, and neutrophils significantly increased in both groups of mice fed with MCD diet regarding control diet, we did not find any significant differences when MCD-fed MyMRKO mice were compared to MCD-fed FC mice (**Supplementary Figure 5A**). Indeed, we only observed a slightly increasing tendency in these myeloid cell populations. Further, the expression of CD86, a costimulatory molecule, was measured on the surface of antigen-presenting cells. Monocyte expression of CD86 increased in both groups of MCD-fed mice as compared to chow-fed control mice (**Supplementary Figure 5B**). However, macrophages from MCD-fed FC mice expressed higher levels of CD86, as compared to controls, while MyMRKO fed with MCD diet failed to increase the expression of this costimulatory molecule in response to MCD feeding (**Supplementary Figure 5C**).

**Figure 6:**
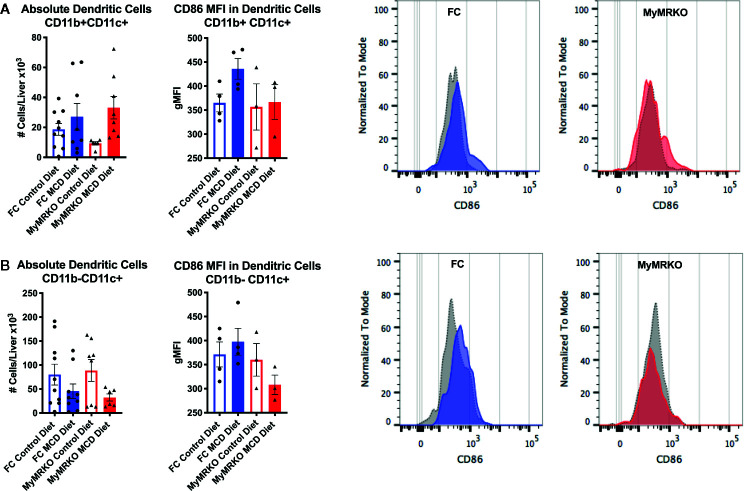
Dendritic cell (DC) subsets in the liver of myleoid mineralocorticoide receptor knockout (MyMRKO) mice display a tendency to express lower CD86 independent of the cell number. Flow cytometry was used to measure the absolute number (left panel) and gMFI expression of *Cd86* of two subsets of DCs: CD11b^+^CD11c^+^ **(A)** and CD11b-CD11c^+^ **(B)**. For each cellular subset a representative image of CD86 expression in floxed control (FC) and MyMRKO fed with chow diet (gray dotted histogram) or MCD diet (colored histogram) is shown. Statistical analysis was performed with one-way ANOVA comparing all treatments, with Tukey post-test. All figures display mean ± SEM. Statistical differences were considered significant according to *p < 0.05.

DC populations were differentially analyzed as CD11b^+^CD11c^+^ and CD11b^-^CD11c^+^ subsets (38). The number of CD11b^+^CD11c^+^ DCs showed a tendency to increase in MCD-fed mice compared to their respective controls (**Figure 6A**). However, despite similar increases in cell numbers, the expression of CD86 was unchanged in MCD-fed MyMRKO mice and increased in MCD-fed FC mice compared to control diet-fed mice. This result suggest that liver-infiltrating DCs from MCD-fed MyMRKO mice could be less effective at presenting antigens with equivalent costimulatory signaling to T cells, due to a lower expression per cells ratio. Additionally, the number of CD11b^-^CD11c^+^ DCs in livers showed a tendency to decrease in both MCD-fed groups (**Figure 6B**), probably due to their mobilization to spleen or lymph nodes for antigen presentation to T cells. The expression of the MHC class II IA/IE molecules was measured on liver-infiltrating DCs (Supplementary **Figure 6**), which showed no significant expression differences, suggesting that a reduced expression was only observed in CD86 molecule.

### MyMRKO DCs Show an Impaired Capacity to Prime CD8^+^ T Cells During an Inflammatory Response

Our data suggest that CD8+ T cells showed a reduced activation (**Figure 5**) and lower migration to the target organ (**Figure 4**), which associates with the steatosis phenotype observed in MCD-fed MyMRKO mice, probably due to low costimulatory capacity. For that reason, we measured the *in vitro* response of DCs to LPS. First, we performed a dose-response curve to define the LPS concentration required to induce maximal activity. **Supplementary Figure 7** shows that at 12 μg/ml of LPS was an optimal concentration to perform these assays. Thus, we decided to perform the following experiments using LPS at 12 and 62 μg/ml. We found that MyMRKO-derived DCs displayed an impaired capacity to express maturation markers and secreted lower levels of IL-12 and high levels of IL-10 in contrast with control FC-derived DCs (**Supplementary Figures 8** and **9**).

**Figure 7:**
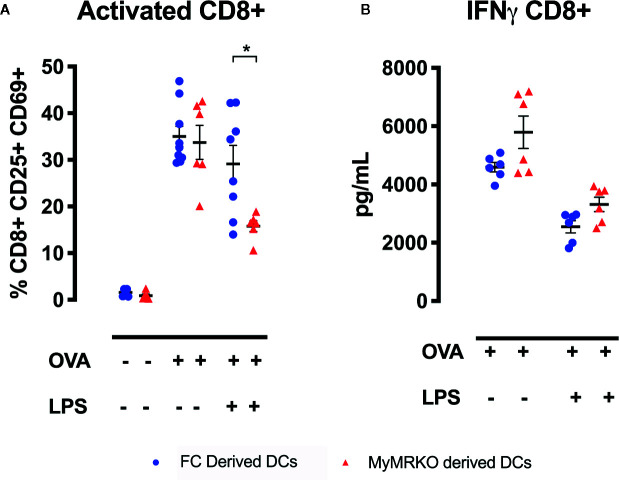
Cocultures between OT-I lymphocytes and bone marrow-derived dendritic cells (DCs) from myleoid mineralocorticoide receptor knockout (MyMRKO) induce lower expression of activation markers CD69 and CD25. Purified CD8^+^ OT-I lymphocytes were cocultured with DCs in a ratio of 1:1 for 48 h. Three different conditions were tested: cocultures with DCs : CD8^+^T lymphocytes without OVA peptide, untreated DCs : CD8^+^T lymphocytes plus OVA peptide, and lipopolysaccharide (LPS)-pretreated DCs : CD8^+^T lymphocytes plus OVA peptide **(A)**. Expression of activation markers CD69 and CD25 in CD8^+^ T lymphocytes **(B)**. IFN-γ secretion in the supernatants by ELISA. Statistical analysis was performed with two-way ANOVA comparing all treatments, with Tukey post-test. All figures display mean ± SEM. Statistical differences were considered significant according to *p < 0.05.

To further define this mechanism, purified transgenic CD8^+^ OT-I T cells were stimulated with antigen (OVAp)-loaded DCs obtained from MyMRKO or FC mice. Although MyMRKO- and FC-derived DCs were equivalent at activating CD8^+^ OT-I T cells (**Figure 7A**), this pattern changed in the presence of an inflammatory stimulus, such as LPS. The frequency of activated CD69^+^CD25^+^CD8^+^ OT-I T cells stimulated with MyMRKO DCs was significantly lower as compared to stimulation with FC DCs (**Figure 7A**). Conversely, no significant differences were observed when IFN-γ secretion was measured for CD8^+^ OT-I T cells stimulated with either OVAp-loaded MyMRKO or FC DCs (**Figure 7B**).

We also measured the response of CD4 T cells, for that transgenic CD4^+^ OT-II T cells were stimulated with MyMRKO or FC DCs, no significant differences were observed for the activation of these cells (**Supplementary Figure 10**). Although IL-10 and IL-17 secretion was similar for all groups, MyMRKO DCs showed a tendency to promote a higher secretion of IFN-γ by CD4^+^ OT-II T cells. However, when DCs were pretreated with LPS as an inflammatory stimulus, FC DCs showed a tendency to a reduced secretion of IFN-γ and IL-10 and increased IL-17, as compared to MyMRKO DCs.

## Discussion

Animal models of NASH include those with genetic mutations, chemical-mediated disease, and diet-induced approaches that alter hepatocyte metabolism (30, 31). Here we used a nutritional deficiency model to evaluate the role of MR in mediating inflammatory response in liver steatosis. MCD diet induces NASH because methionine and choline are required for hepatic secretion of triglycerides in the form of very low-density lipoproteins (VLDL). Lack of these nutrients results in impaired lipid exportation from liver to peripheral tissues, producing a phenotype of macrovesicular liver steatosis, hepatocellular death, inflammation, oxidative stress, and mild fibrosis (39). We observed a body weight loss of ~25% in MCD-fed mice, which was expected due to hippophage and hyper-catabolism described for that type of diet (31).

Reduced steatosis phenotype observed in MCD-fed MyMRKO mice indicates that the MR in myeloid cells is involved in modulating lipid accumulation. Similar results were reported in an obese mouse model with MR deficiency (*Lep^ob/ob^* crossed with MyMRKO), in which MR absence on KCs impaired their crosstalk with hepatocytes, but in a context of insulin resistance (29). In that study, obese female MyMRKO mice showed improved insulin sensitivity and glucose homeostasis and lower liver steatosis as compared to FC and hepatocytes from MyMRKO mice were reported to exhibit downregulated expression of genes involved in lipogenesis and lipid storage, such as, *SCD-1, Ly6d*, and *Cidea* (29). Of note, studies assessing the effects of pharmacologic MR antagonism using spironolactone or eplerenone (27, 28) have shown that MR antagonism prevents liver steatosis in animal models of NASH, reinforcing the role of MR in modulating steatosis development.

The well documented role that the MR and RAAS play in fibrosis and end-organ damage during hypertension has prompted research to explore MR antagonism as a new therapy for NASH (40). Here we showed that, despite lower steatosis in MCD-fed MyMRKO mice, the histological and molecular features of fibrosis remain unaffected by MR deletion in myeloid cells (including higher levels of profibrotic markers *Col1A, Mmp-2, Timp-1, and Tgfβ*). In previous studies of MR antagonism in NASH, liver fibrosis was prevented or reduced (27, 28). This difference may result from systemic effects, because MyMRKO mice lack MR only on myeloid cells, while pharmacologic MR antagonism effects all cells expressing MR. Similarly, to previously published models, in the present study mice showed high levels of serum aldosterone (**Figure 3**). However, in our model fibrosis was not prevented. This finding may reflect that in the liver, other cell types, such as hepatocytes and endothelial cells, express MR and respond to aldosterone stimulation, secreting profibrotic and proinflammatory factors (41). Consistently, mouse models for induced hypertension in which MR is lacking (MyMRKO) (26) showed reduced cardiac fibrosis as compared to control hypertensive mice. *In vitro* analyses performed in peritoneal macrophages showed that an MR deficiency promoted macrophages polarization toward a M2 phenotype, decreasing the expression of fibrotic markers (26).

The establishment of chronic inflammation is crucial for NASH development (42). We found that mice fed with MCD diet displayed significant leukocyte infiltration and increased levels of proinflammatory cytokines *Il-18* (**Supplementary Figure 2**), underscoring the establishment of an inflammatory environment. In addition, *Tnf-α* and *Il-1β* levels increased in both MCD-fed FC and MyMRKO mice (26). A previous report found that a similar phenotype of liver steatosis did not induce significant changes in inflammatory mediators, such as *Il-6, Tnf-α*, or *Il-1β* (29).

Analyses of human livers revealed that patients with NAFLD accumulate CD8^+^ T cells with enhanced expression of interferon regulatory factors (IRFs) and interferon stimulatory genes (ISGs), which correlate with the NAS score of patients (43). Therefore, fatty livers exhibit elevated IFN-I responses and CD8^+^ T cell infiltration, serving as potential biomarkers of disease (43). Additional evidence about the role of CD8^+^ T cells in NASH has been generated in animal models either lacking these cells (37, 38) or effector molecules, such as IFNγ (44) or perforin (18). In all above-mentioned models, steatosis was prevented when the function of CD8^+^ T cells was deficient. Here, we proposed that an MR-deficiency in myeloid cells can influence the infiltration of CD8^+^ T cell to the liver in a NASH animal model.

It is important to note that, in this study, we made modifications to the tissue disaggregation and flow cytometry procedure described by VanSaun, M.N. et al. (33). In this work, the authors showed that the percentage of CD8+ T cells found in livers is lower than CD4+ T cells. In a previous work that we used the same protocol but in an infectious animal model of Salmonella enterica, we also found a similar ratio between CD4+ and CD8+ T cells in livers (45). Both studies agree with the results displayed in **Figure 4**, in which the number and percentage of CD8+ T cells analyzed by flow cytometry are lower than CD4+ T cells.

Despite a lack of direct evidence linking high levels of aldosterone to altered CD8^+^ T cell function in NASH, two studies performed in patients with primary aldosteronism demonstrated that high aldosterone levels are correlated with NAFLD development (46). In addition, a significant association was reported between high levels of aldosterone and fatty liver in HIV patients (47), who have higher numbers of CD8^+^ T cells.

CD25 and CD69 activation markers measured in CD8+ T cells after antigen activation indicated their capacity to proliferates in responds to IL-2 and its ability to migrate to target organ, respectively (48). However, cytokine milieu can promote per se and antigen-independent fashion the secretion of IFN-γ for CD8+ T cell. In this line, it has been reported that IL-10 can act as inhibitory cytokine in CD8+ T cell activation when IL-12 is present, but in the presence of IL-18, IL-10 enhance the IFN-γ secretion by CD8+ T lymphocytes (49). This work agrees with our results displayed in **Supplementary Figure 9**, in which MyMRKO DCs stimulated with LPS secretes higher levels of IL-10 and lower levels of IL-12 p70 than controls. Despite we did not measure IL-18 levels in coculture supernatants, RT-PCRs performed in livers high levels of IL-18 in MCD-fed mice (**Supplementary Figure 2**). In addition, direct stimulation of TLR ligands (such as LPS) can elicit effector CD8+ T cells to produce IFN-γ and modulates its accumulation in several lymphoid organs (50). We mention this point, because is only in the coculture with LPS matured DCs that differences in CD8+ T cell activation is seen. Although we did not measure the CTLA-4 expression in CD8+ T cell, it has been described that self-regulation of CD8+T cell function is mediated by DCs CD80/CD86 engagement to this molecule after antigen stimulation. As a result, the cytokine profile are modulated (mainly IFN-γ production) rather than IL-2 mediated CD8+ T cell proliferation and activation (51). In our context, we found that MyMRKO DCs pulsed with LPS displayed significant lower expression in CD86 (**Supplementary Figure 8A**), which can be linked with the non-altered levels in IFN-γ secretion. However, there is many other effector molecules such as granzyme and perforin, and cytolytic or regulatory mechanism to be investigated (52).

It is important to highlight that signaling pathways involved in cell maturation and cytokine secretion may also be altered by the absence of MR in myeloid cells, because one of the nongenomic effects of MR is acting as second messenger (25). For all described reasons, many questions are still open, and further and deeper analysis are required to understand how the lack of MR in myeloid cells influenced specifically the profile of activation and migration of CD8+ T cells described in this work.

Our data suggest a model in which MyMRKO mice fed with MCD diet display lower steatosis *via* inflammatory response that crosstalk with hepatocytes. Simultaneously, fibrosis appears to involve hepatic cells rather than myeloid cells. Therefore, we postulate that high levels of plasmatic aldosterone could act through MR expressed by hepatocytes, HSC or endothelial cells to induce fibrosis.

One of the most significant limitations of our study is the MCD feeding model, because mice did not developed metabolic syndrome features, insulin resistance nor obesity. However, the MCD mouse model—as shown in **Supplementary Figure 1**—suffers a transient but pronounced weight loss during the first two weeks after feeding. This change is attributed to an increased sympathetic outflow to adipose tissue, which can lead to a reduction in the energy extraction efficiency from nutrients *via* high mitochondrial uncoupling (53, 54). We choose the MCD model to avoid the influence of the extrahepatic disorders that occur concomitantly with NAFLD development (55). Another limitation of four study is the gender of the animals used, because of the difficulty to obtaining enough conditional knockout mice, we decided to perform experiments in both males and female mice. It was previously shown that female mice are more susceptible to suffering steatosis than males while males developed more inflammation (56). Despite of this, separate gender analyses showed no significant differences between females and males in our study (data not shown). This finding is supported by the fact that most of the observations regarding gender differences were made in obesogenic, atherogenic, and insulin resistant animal models (55). In addition, while studies have shown that estrogen plays a role at protecting from liver injury following a high-fat diet (57), absence of protection was observed in MCD diet-induced steatohepatitis experiments in female mice that received an ovariectomy or antiestrogen treatment (58). Furthermore, another study showed that female mice fed a chow diet supplemented with 30% fructose in the drinking water can display a more pronounced inflammatory response than male mice (55).

In summary, our data suggest that the development of steatosis requires aldosterone stimulation through MR in antigen-presenting cells that direct the CD8+ T cell lymphocyte response. This response establishes a proinflammatory environment with greater IFN-γ secretion in the liver, affecting hepatocyte lipid accumulation. However, further investigation is necessary to understand the impaired capacity of antigen-presenting cells to stimulate CD8+ T cells in MyMRKO mice, as well as the effect of aldosterone *via* hepatocytes or HSC to maintain a fibrotic phenotype.

## Data Availability Statement

The raw data supporting the conclusions of this article will be made available by the authors, without undue reservation.

## Ethics Statement

The animal study was reviewed and approved by Comité de Ética y Bienestar Animal (CEBA–UC) Pontificia Universidad Católica de Chile (CEBA #170525006).

## Author Contributions

NM-D, DC, EJ, and AH: Performed research. NM-D and DC: Analyzed data. MA, AK, NM-D and DC: Designed research. MA, AK, NM-D, and DC: Wrote the manuscript. MA and AK: Edited the paper. All authors contributed to the article and approved the submitted version.

## Funding

The work of the authors was supported by the Chilean government through the Fondo Nacional de Desarrollo Científico y Tecnológico (FONDECYT 1190830 to AK, 1191145 to MA, and 11171001 to DC), the Comisión Nacional de Investigación Científica y Tecnológica (grant CONICYT PIA/Basal PFB12, Basal Centre for Excellence in Science and Technology to MA), Institute on Immunology and Immunotherapy P09/016-F ICN09_016 to AK. NM-D was a CONICYT Fellow 21140178.

## Conflict of Interest

The authors declare that the research was conducted in the absence of any commercial or financial relationships that could be construed as a potential conflict of interest.

## Abbreviations

CDAA, choline deficient, L-amino acid defined diet; DAMPs, amage-associated molecular pattern; DCs, dendiritic cells; FC, floxed-control mice; gMFI, geometric mean; HCC, hepatocellular carcinoma; HGF, hepatocyte growth factor; HSC, hepatic stellate cell; HTG, hepatic glycerides; H/E, Hematoxylin-eosine and immunohistochemical staining of…; KCs, Kupffer cells; LPS, lipopolysaccharides; MCD, methionie and choline deficient diet; MR, mineralocorticoid receptor; MyMRKO, myleoid mineralocorticoide receptor; NAFLD, non-alcoholic fatty liver disease; NAS, NAFLD activity score; NASH, non-alcoholic steatohepatitis; RAAS, Renin-Angiotensin-Aldosterone System (RAAS); VLDL, very low density lipoprotein.

## References

[1] Cotter TG Rinella M Nonalcoholic Fatty Liver Disease 2020: The State of the Disease. Gastroenterology (2020) 158:1851–64. 10.1053/j.gastro.2020.01.052 32061595

[2] Younossi Z Tacke F Arrese M Chander Sharma B Mostafa I Bugianesi E Global Perspectives on Nonalcoholic Fatty Liver Disease and Nonalcoholic Steatohepatitis. Hepatology (2019) 69:2672–82. 10.1002/hep.30251 30179269

[3] Arab JP Arrese M Trauner M Recent Insights into the Pathogenesis of Nonalcoholic Fatty Liver Disease. Annu Rev Pathol Mech Dis (2018) 13:321–50. 10.1146/annurev-pathol-020117-043617 29414249

[4] Bedossa P Pathology of non-alcoholic fatty liver disease. Liver Int (2017) 37(Suppl 1):85–9. 10.1111/liv.13301 28052629

[5] Vernon G Baranova A Younossi ZM Systematic review: the epidemiology and natural history of non-alcoholic fatty liver disease and non-alcoholic steatohepatitis in adults. Aliment Pharmacol Ther (2011) 34:274–85. 10.1111/j.1365-2036.2011.04724.x 21623852

[6] Schuster S Cabrera D Arrese M Feldstein AE Triggering and resolution of inflammation in NASH. Nat Rev Gastroenterol Hepatol (2018) 15:349–64. 10.1038/s41575-018-0009-6 29740166

[7] Puche JE Lee YA Jiao J Aloman C Fiel MI Muñoz U A novel murine model to deplete hepatic stellate cells uncovers their role in amplifying liver damage in mice. Hepatology (2013) 57:339–50. 10.1002/hep.26053 PMC3522764 22961591

[8] Yu M-C Chen C-H Liang X Wang L Gandhi CR Fung JJ Inhibition of T-cell responses by hepatic stellate cells *via* B7-H1–mediated T-cell apoptosis in mice. Hepatology (2004) 40:1312–21. 10.1002/hep.20488 15565659

[9] Arrese M Cabrera D Kalergis AM Feldstein AE Innate Immunity and Inflammation in NAFLD/NASH. Dig Dis Sci (2016) 61:1294–303. 10.1007/s10620-016-4049-x PMC4948286 26841783

[10] Nobili V Parola M Alisi A Marra F Piemonte F Mombello C Oxidative stress parameters in paediatric non-alcoholic fatty liver disease. Int J Mol Med (2010) 26:471–6. 10.3892/ijmm_00000487 20818484

[11] Heier E-C Meier A Julich-Haertel H Djudjaj S Rau M Tschernig T Murine CD103^+^ dendritic cells protect against steatosis progression towards steatohepatitis. J Hepatol (2017) 66:1241–50. 10.1016/j.jhep.2017.01.008 28108233

[12] Alkhouri N Morris-Stiff G Campbell C Lopez R Tamimi TA-R Yerian L Neutrophil to lymphocyte ratio: a new marker for predicting steatohepatitis and fibrosis in patients with nonalcoholic fatty liver disease. Liver Int (2012) 32:297–302. 10.1111/j.1478-3231.2011.02639.x 22097893

[13] Zhou Z Xu M-J Cai Y Wang W Jiang JX Varga ZV Neutrophil–Hepatic Stellate Cell Interactions Promote Fibrosis in Experimental Steatohepatitis. Cell Mol Gastroenterol Hepatol (2018) 5:399–413. 10.1016/j.jcmgh.2018.01.003 29552626 PMC5852390

[14] Sutti S Albano E Adaptive immunity: an emerging player in the progression of NAFLD. Nat Rev Gastroenterol Hepatol (2020) 17:81–92. 10.1038/s41575-019-0210-2 31605031 PMC7222953

[15] Van Herck MA Weyler J Kwanten WJ Dirinck EL De Winter BY Francque SM The Differential Roles of T Cells in Non-alcoholic Fatty Liver Disease and Obesity. Front Immunol (2019) 10:82. 10.3389/fimmu.2019.00082 30787925 PMC6372559

[16] Sutti S Jindal A Locatelli I Vacchiano M Gigliotti L Bozzola C Adaptive immune responses triggered by oxidative stress contribute to hepatic inflammation in NASH. Hepatology (2014) 59:886–97. 10.1002/hep.26749 24115128

[17] Tang Y Bian Z Zhao L Liu Y Liang S Wang Q Interleukin-17 exacerbates hepatic steatosis and inflammation in non-alcoholic fatty liver disease. Clin Exp Immunol (2011) 166:281–90. 10.1111/j.1365-2249.2011.04471.x PMC3219903 21985374

[18] Wang T Sun G Wang Y Li S Zhao X Zhang C The immunoregulatory effects of CD8 T-cell–derived perforin on diet-induced nonalcoholic steatohepatitis. FASEB J (2019) 33:8490–503. 10.1096/fj.201802534RR 30951375

[19] Paschos P Tziomalos K Nonalcoholic fatty liver disease and the renin-angiotensin system: Implications for treatment. World J Hepatol (2012) 4:327. 10.4254/wjh.v4.i12.327 23355909 PMC3554795

[20] Queisser N Happ K Link S Jahn D Zimnol A Geier A Aldosterone induces fibrosis, oxidative stress and DNA damage in livers of male rats independent of blood pressure changes. Toxicol Appl Pharmacol (2014) 280:399–407. 10.1016/j.taap.2014.08.029 25204689

[21] Bataller R Sancho-bru P Ginès P Lora JM Al-garawi A Solé M Activated human hepatic stellate cells express the renin-angiotensin system and synthesize angiotensin II. Gastroenterology (2003) 125:117–25. 10.1016/S0016-5085(03)00695-4 12851877

[22] Herrada AA Contreras FJ Marini NP Amador CA González PA Cortés CM Aldosterone Promotes Autoimmune Damage by Enhancing Th17-Mediated Immunity. J Immunol (2010) 184:191–202. 10.4049/jimmunol.0802886 19949098

[23] Muñoz-Durango N Vecchiola A Gonzalez-Gomez LM Simon F Riedel CA Fardella CE Modulation of Immunity and Inflammation by the Mineralocorticoid Receptor and Aldosterone. BioMed Res Int (2015) 2015:14. 10.1155/2015/652738. PMC4581510 26448944

[24] Amador CA Barrientos V Peña J Herrada AA González M Valdés S Spironolactone Decreases DOCA–Salt–Induced Organ Damage by Blocking the Activation of T Helper 17 and the Downregulation of Regulatory T Lymphocytes. Hypertension (2014) 63:797–803. 10.1161/HYPERTENSIONAHA.113.02883 24420551

[25] Munoz-Durango N Barake MF Letelier NA Campino C Kalergis CEF and AM. Immune System Alterations by Aldosterone During Hypertension: From Clinical Observations to Genomic and Non-Genomic Mechanisms Leading to Vascular Damage. Curr Mol Med (2013) 13:1035–46. 10.2174/1566524011313060015 23590758

[26] Usher MG Duan SZ Ivaschenko CY Frieler RA Berger S Schütz G Myeloid mineralocorticoid receptor controls macrophage polarization and cardiovascular hypertrophy and remodeling in mice. J Clin Invest (2010) 120:3350–64. 10.1172/JCI41080 PMC2929712 20697155

[27] Wada T Miyashita Y Sasaki M Aruga Y Nakamura Y Ishii Y Eplerenone ameliorates the phenotypes of metabolic syndrome with NASH in liver-specific SREBP-1c Tg mice fed high-fat and high-fructose diet. Am J Physiol Metab (2013) 305:E1415–25. 10.1152/ajpendo.00419.2013 24129399

[28] Pizarro M Solís N Quintero P Barrera F Cabrera D Rojas-de Santiago P Beneficial effects of mineralocorticoid receptor blockade in experimental non-alcoholic steatohepatitis. Liver Int (2015) 35:2129–38. 10.1111/liv.12794 PMC4522413 25646700

[29] Zhang Y-Y Li C Yao G-F Du L-J Liu Y Zheng X-J Deletion of Macrophage Mineralocorticoid Receptor Protects Hepatic Steatosis and Insulin Resistance Through ERα/HGF/Met Pathway. Diabetes (2017) 66:1535–47. 10.2337/db16-1354 PMC5860190 28325853

[30] Jahn D Kircher S Hermanns HM Geier A Animal models of NAFLD from a hepatologist’s point of view. Biochim Biophys Acta - Mol Basis Dis (2019) 1865:943–53. 10.1016/j.bbadis.2018.06.023 29990551

[31] Hansen HH Feigh M Veidal SS Rigbolt KT Vrang N Fosgerau K Mouse models of nonalcoholic steatohepatitis in preclinical drug development. Drug Discovery Today (2017) 22:1707–18. 10.1016/J.DRUDIS.2017.06.007 28687459

[32] Kleiner DE Brunt EM Van Natta M Behling C Contos MJ Cummings OW Design and validation of a histological scoring system for nonalcoholic fatty liver disease. Hepatology (2005) 41:1313–21. 10.1002/hep.20701 15915461

[33] VanSaun MN Mendonsa AM Lee Gorden D Hepatocellular Proliferation Correlates with Inflammatory Cell and Cytokine Changes in a Murine Model of Nonalchoholic Fatty Liver Disease. PloS One (2013) 8:e73054. 10.1371/journal.pone.0073054 24039859 PMC3767686

[34] Hung C-S Chou C-H Liao C-W Lin Y-T Wu X-M Chang Y-Y Aldosterone Induces Tissue Inhibitor of Metalloproteinases-1 Expression and Further Contributes to Collagen Accumulation. Hypertension (2016) 67:1309–20. 10.1161/HYPERTENSIONAHA.115.06768 27113051

[35] Hermoso MA Matsuguchi T Smoak K Cidlowski JA Glucocorticoids and Tumor Necrosis Factor Alpha Cooperatively Regulate Toll-Like Receptor 2 Gene Expression. Mol Cell Biol (2004) 24:4743–56. 10.1128/MCB.24.11.4743-4756.2004 PMC416411 15143169

[36] Stojsavljević S Gomerčić Palčić M Virović Jukić L Smirčić Duvnjak L Duvnjak M Adipokines and proinflammatory cytokines, the key mediators in the pathogenesis of nonalcoholic fatty liver disease. World J Gastroenterol (2014) 20:18070–91. 10.3748/wjg.v20.i48.18070 PMC4277948 25561778

[37] Bhattacharjee J Kirby M Softic S Miles L Salazar-Gonzalez R-M Shivakumar P Hepatic natural killer T-cell and CD8+ T-cell signatures in mice with nonalcoholic steatohepatitis. Hepatol Commun (2017) 1:299–310. 10.1002/hep4.1041 29152605 PMC5687094

[38] Wolf MJ Adili A Piotrowitz K Abdullah Z Boege Y Stemmer K Metabolic activation of intrahepatic CD8+ T cells and NKT cells causes nonalcoholic steatohepatitis and liver cancer *via* cross-talk with hepatocytes. Cancer Cell (2014) 26:549–64. 10.1016/j.ccell.2014.09.003 25314080

[39] Caballero F Fernández A Matías N Martínez L Fucho R Elena M Specific contribution of methionine and choline in nutritional nonalcoholic steatohepatitis: impact on mitochondrial S-adenosyl-L-methionine and glutathione. J Biol Chem (2010) 285:18528–36. 10.1074/jbc.M109.099333 PMC2881778 20395294

[40] Muñoz-Durango N Fuentes C Castillo A González-Gómez L Vecchiola A Fardella C Role of the Renin-Angiotensin-Aldosterone System beyond Blood Pressure Regulation: Molecular and Cellular Mechanisms Involved in End-Organ Damage during Arterial Hypertension. Int J Mol Sci (2016) 17:797. 10.3390/ijms17070797 PMC4964362 27347925

[41] Jia G Habibi J Aroor AR Martinez-Lemus LA DeMarco VG Ramirez-Perez FI Endothelial Mineralocorticoid Receptor Mediates Diet-Induced Aortic Stiffness in Females. Circ Res (2016) 118:935–43. 10.1161/CIRCRESAHA.115.308269 PMC4798906 26879229

[42] Sutti S Jindal A Bruzzì S Locatelli I Bozzola C Albano E Is there a role for adaptive immunity in nonalcoholic steatohepatitis? World J Hepatol (2015) 7:1725. 10.4254/wjh.v7.i13.1725 26167244 PMC4491900

[43] Ghazarian M Revelo XS Nøhr MK Luck H Zeng K Lei H Type I interferon responses drive intrahepatic T cells to promote metabolic syndrome. Sci Immunol (2017) 2:eaai7616. 10.1126/sciimmunol.aai7616 28567448 PMC5447456

[44] Luo X-Y Takahara T Kawai K Fujino M Sugiyama T Tsuneyama K IFN-γ deficiency attenuates hepatic inflammation and fibrosis in a steatohepatitis model induced by a methionine- and choline-deficient high-fat diet. Am J Physiol Liver Physiol (2013) 305:G891–9. 10.1152/ajpgi.00193.2013 24136786

[45] Salazar GA Peñaloza HF Pardo-Roa C Schultz BM Muñoz-Durango N Gómez RS Interleukin-10 Production by T and B Cells Is a Key Factor to Promote Systemic Salmonella enterica Serovar Typhimurium Infection in Mice. Front Immunol (2017) 8:889. 10.3389/fimmu.2017.00889 28824622 PMC5539121

[46] Taylor RS Ashton KE Moxham T Hooper L Ebrahim S Reduced Dietary Salt for the Prevention of Cardiovascular Disease: A Meta-Analysis of Randomized Controlled Trials (Cochrane Review). Am J Hypertens (2011) 24:843–53. 10.1038/ajh.2011.115 21731062

[47] Srinivasa S Fitch KV Quadri N Maehler P O’Malley TK Martinez-Salazar EL Significant Association of Aldosterone and Liver Fat Among HIV-Infected Individuals With Metabolic Dysregulation. J Endocr Soc (2018) 2:1147–57. 10.1210/js.2018-00194 PMC6162603 30283827

[48] Cibrián D Sánchez-Madrid F CD69: from activation marker to metabolic gatekeeper. Eur J Immunol (2017) 47:946–53. 10.1002/eji.201646837 PMC6485631 28475283

[49] Freeman BE Hammarlund E Raué H-P Slifka MK Regulation of innate CD8+ T-cell activation mediated by cytokines. Proc Natl Acad Sci (2012) 109:9971–6. 10.1073/PNAS.1203543109 PMC3382521 22665806

[50] Liu W Menoret A Vella AT Responses to LPS boost effector CD8 T-cell accumulation outside of signals 1 and 2. Cell Mol Immunol (2017) 14:254–64. 10.1038/cmi.2015.69 PMC5360877 26189366

[51] Pandiyan P Hegel JKE Krueger M Quandt D Brunner-Weinzierl MC High IFN-γ Production of Individual CD8 T Lymphocytes Is Controlled by CD152 (CTLA-4). J Immunol (2007) 178:2132–40. 10.4049/jimmunol.178.4.2132 17277117

[52] Hosking MP Flynn CT Whitton JL Antigen-Specific Naive CD8&lt;sup<+&lt;/sup< T Cells Produce a Single Pulse of IFN-γ In Vivo within Hours of Infection, but without Antiviral Effect. J Immunol (2014) 193(4):1873–85. 10.4049/jimmunol.1400348 PMC4119517 25015828

[53] Machado MV Michelotti GA Xie G de Almeida TP Boursier J Bohnic B Mouse Models of Diet-Induced Nonalcoholic Steatohepatitis Reproduce the Heterogeneity of the Human Disease. PloS One (2015) 10:e0127991. 10.1371/journal.pone.0127991 26017539 PMC4446215

[54] Rizki G Arnaboldi L Gabrielli B Yan J Lee GS Ng RK Mice fed a lipogenic methionine-choline-deficient diet develop hypermetabolism coincident with hepatic suppression of SCD-1. J Lipid Res (2006) 47:2280–90. 10.1194/jlr.M600198-JLR200 16829692

[55] Spruss A Henkel J Kanuri G Blank D Püschel GP Bischoff SC Female Mice Are More Susceptible to Nonalcoholic Fatty Liver Disease: Sex-Specific Regulation of the Hepatic AMP-Activated Protein Kinase-Plasminogen Activator Inhibitor 1 Cascade, but Not the Hepatic Endotoxin Response. Mol Med (2012) 18:1346–55. 10.2119/molmed.2012.00223 PMC3521787 22952059

[56] Ganz M Csak T Szabo G High fat diet feeding results in gender specific steatohepatitis and inflammasome activation. World J Gastroenterol (2014) 20:8525. 10.3748/wjg.v20.i26.8525 25024607 PMC4093702

[57] Kamada Y Kiso S Yoshida Y Chatani N Kizu T Hamano M Estrogen deficiency worsens steatohepatitis in mice fed high-fat and high-cholesterol diet. Am J Physiol Liver Physiol (2011) 301:G1031–43. 10.1152/ajpgi.00211.2011 21885686

[58] Kashireddy PRV Rao MS Sex Differences in Choline-Deficient Diet-Induced Steatohepatitis in Mice. Exp Biol Med (2004) 229:158–62. 10.1177/153537020422900204 14734794

