# Supplementary material for: A Mineralocorticoid Receptor Deficiency in Myeloid Cells Reduces Liver Steatosis by Impairing Activation of CD8+ T Cells in a Nonalcoholic Steatohepatitis Mouse Model
Source: Front Immunol. 2020 Dec 16;11:563434. doi: 10.3389/fimmu.2020.563434 (PMC7772468; doi:10.3389/fimmu.2020.563434)
Supplement: Supplementary file 1 [file DataSheet_1.pdf]

Supplementary Information for

A deficiency of mineralocorticoid receptor in myeloid cells reduces liver steatosis by impairing activation of CD8+ T cells in a nonalcoholic steatohepatitis mouse model

Natalia Muñoz-Durango[1], Marco Arrese[2,3], Alejandra Hernández[2], Evelyn Jara[1,4], Alexis M. Kalergis[1,5,*] and Daniel Cabrera[2,6,*].

[1]Millennium Institute on Immunology and Immunotherapy. Departamento de Genética Molecular y Microbiología, Facultad de Ciencias Biológicas. Pontificia Universidad Católica de Chile.

[2]Departamento de Gastroenterología, Escuela de Medicina, Pontificia Universidad Catolica de Chile.

[3]Centro de Envejecimiento y Regeneración (CARE), Departamento de Biología Celular y Molecular, Facultad de Ciencias Biológicas Pontificia Universidad Católica de Chile, Santiago, Chile

[4]Departamento de Ciencias Básicas, Facultad de Ciencias, Universidad Santo Tomás, Chile.

[5]Departamento de Endocrinología, Facultad de Medicina, Pontificia Universidad Católica de Chile.

[6]Facultad de Ciencias Médicas, Universidad Bernardo O Higgins, Santiago, Chile.

[*]Corresponding authors: Daniel Cabrera and Alexis M. Kalergis.

Email: dacabrer@puc.cl; akalergis@bio.puc.cl; akalergis@icloud.com

This PDF file includes:

Supplementary methods

Figures S1 to S10

Table S1

Supplementary materials and methods.

Genotyping of MyMRO Mice

To determinate whether the genotype of mice corresponded to MyMRKO or its Floxed Control (FC), all mice were genotyped by PCR from peripheral blood samples with the following set of primers: Cre-Fw 5'-AGG TGT AGA GAA GGC ACT CAG C-3', Cre-Rv 5'-CTA ATC GCC ATC TTC CAG CAG G-3', the product size was 411 bp and should be present only in Cre+ mice. The control gene was MEH-Fw 5'-AAG TGA GTT TGC ATG GCG CAG C-3', MEH-Rv 5'-CCC TTT AGC CCC TTC CCT CTG-3', the product size was 341 bp and should be present in all animals. Primer A (MRflox) 5'-CTGGAGATCTGAACTCCAGGCT-3', Primer B (MRflox) 5'-TAG AAA CAC TTC GTA AAG TAG AGC T-3', Primer C (MRflox) 5'-CCT AGA GTT CCT GAG CTG CTG A-3', the product sizes were for the WT allele: 285 bp, for the Floxed allele: 335 bp, and for the Null allele: 390 bp. The PCR amplification program for Cre and MEH genes encompassed: one cycle of incubation at 95ºC for 5 minutes, followed by 35 cycles of denaturation 95ºC during 30 seconds / annealing 55ºC during 30 seconds / extension 72ºC for 45 seconds and finalizing with 72ºC for 7 minutes. The PCR amplification program for Floxed MR gene encompassed: one cycle of incubation at 95ºC for 5 minutes, followed by 35 cycles of denaturation 95ºC during 30 seconds / annealing 63ºC during 60 seconds / extension 72ºC for 60 seconds and finalizing with 72ºC for 60 seconds.

Quantification of Lipid Content

Hepatic triglycerides content (HTC) was quantified from fresh liver samples according manufacturer´s instructions (Human Gesselheit, Wiesbaden, Germany).

Flow cytometric stain and gating strategy

Liver infiltrated leukocytes were divided in three aliquots to be stained with the following antibody combinations: 1) Myeloid staining: anti-CD45 PerCP or FITC (clone 30-F11; BD Biosciences), anti-CD11b PE or PECy7 (clone M1/70; BD Biosciences), anti-

CD11c (clone HL3; BD Biosciences), anti-Ly6C PECy7 (clone AL-21; BD Biosciences), anti-Ly6G APC (clone 1A8 ; BD Biosciences), anti-F4/80 FITC (clone BM8; Biolegend); anti-IA/IE APCCy7 (clone M5/114.15.2; BD Bioscience), anti-CD86 APC (clone GL1; BD Bioscience 558539) and anti-CD40 (clone 3/23; BD Bioscience). 2) Lymphoid staining: anti-TCR⍰ PECy7 (clone H57-597; BD Bioscience), anti-CD4 PerCPCy5.5 (clone RM4-5; BD Bioscience), anti-CD8 PE (clone 53-6.7; BD Bioscience), anti-B220 APCCy7 (clone RA3-6B2; BD Bioscience) and anti-CD25 APC (clone PC61; Biolegend). All antibodies were used according to manufacturer guidelines. Finally, for absolute cell counting, we added to each tube, just before collection, 50 μL of CountBrightTM absolute counting Beads (Life Technologies, Oregon, USA). In parallel, spleens were mechanically disrupted and filtered using a 70-μM cell strainer. Cells were recovered by centrifugation and incubated with red blood cells lysis buffer, for 5 min at room temperature. Then, cells were washed twice with PBS and resuspended in PBS with 2% of Fetal Bovine Serum (FBS; Life Technologies). Cells were counted in Neubauer chamber and 1x10^6 cells were used to be stained with appropriate antibody combinations. For intracellular staining, 1x10^6 cells were cultured for 12 hours in the presence of Cell Stimulation Cocktail plus protein transport inhibitors (eBioscience 00-4975-93). Then, cells were stained with anti-TCRβ PECy7 (clone H57-597; BD Bioscience), anti-CD4 FITC (clone RM4-5; BD Bioscience), anti-CD8 PerCP (clone 53-6.7; BD Bioscience), anti-IL-10 APC (clone JES5-16E3; BD Bioscience), anti-IFN-γ (clone XMG1.2; BD Bioscience), and IL-17A (clone TC11-18H10; BD Bioscience). For intracellular staining, cells were fixed and permeabilized with BD Cytofix/Cytoperm (BD Bioscience) followed by the intracellular antibodies staining. Then, each sample was auto- controlled by fluorescence minus one (FMO) strategy.

Finally, all samples were acquiring in BD FACSCanto II (BD Biosciences), and all data were analyzed in FlowJo VX0.7. (BD Biosciences) as described in Supplementary Figure 2. Briefly, Myeloid cells were analyzed and grouped according to their surface expression markers as monocytes (CD45+/CD3-/CD11b+/Ly6Chi/F4/80-), macrophages and Kupffer cells (CD45+/CD3-/CD11b-/Ly6C-/F4/80+), neutrophils (CD45+/CD3-/CD11b+/Ly6Clow/Ly6Ghi), and two sub-populations of dendritic cells (DCs) (CD45+/CD3-/CD11b+/CD11c+ or CD45+/CD3-/CD11b-/CD11c+). Lymphoid cells were measured following the markers of T cells (CD45+/CD3+/CD4+ and / or CD8+) and markers of B cells CD45+/CD3-/B220+.

Cocultures and Cytokine Secretion by ELISAs

DCs were derived from bone marrow of C57BL/6, MyMRKO, and FC mice, as previously described with some modifications (1). Briefly, tibias and femurs were aseptically removed from mice and bone marrow was obtained by mechanical rinse with sterile PBS using a syringe inside of the bone. Precursor cells were collected and centrifuged for 5 minutes at 300 g at 4°C. Pellet was resuspended in 5 mL of ACK lysis buffer, incubated during 2 minutes at room temperature followed by adding 10 mL of PBS and centrifugation for 5 minutes at 300g at 4°C. Finally, the pellet was washed twice with sterile PBS and resuspended at 1–1,5 $x10^6$ cells/mL in RPMI 1640 culture media (Thermo Fisher Scientific, NY, USA), supplemented with 5% fetal bovine serum (Thermo Fisher Scientific, NY, USA), 100 I.U./mL penicillin/100 μg/mL streptomycin (Thermo Fisher Scientific, NY, USA), 2 mM glutamine (Thermo Fisher Scientific, NY), 1 mM non-essential amino acids (Thermo Fisher Scientific, NY, USA), 1 mM pyruvate (Thermo Fisher Scientific, NY, USA), 1 mM HEPES (Thermo Fisher Scientific, NY), 50 mM β-mercaptoethanol (Winkler, USA), and 10 ng/mL of recombinant murine GM-CSF (Peprotech, NJ, USA). Then 1 mL of cell suspension were seeded in 24-well plates (SP Life Sciences, Korea) and incubated at 37°C with 5% CO2. Culture medium was replaced at 2nd and 4th day post culture, but at 4th day the culture media was supplemented with 5% of charcoal stripped fetal bovine serum (Thermo Fisher Scientific, NY) to avoid steroidal hormones conditioning at the time of aldosterone pulse. At 5th day of differentiation DCs were used for all the experiments. The efficiency of DCs differentiation were determined by analyzing the expression of the specific surface marker CD11c (clone HL3, BD Pharmingen) and IA/IE (clone M5/114.15.2; BD Bioscience) by flow cytometry. DCs were divided pretreated with LPS were stimulated during 24 hours with LPS from Salmonella enterica serotype Typhimurium (Sigma-Aldrich) at 50 pg/mL (2) . T lymphocytes were enriched by sorting (BD FACSAria II) from OT-I or OT-II splenocytes. Then, cells were counted and plated at $1x10^5$ cell in 100 μL of each cocultured cells, to achieve final volume of 200 uL with a final cell ratio 1:1 (Falcon™, 96 wells round bottom). Three different conditions were tested: cocultures with DCs: CD4+ or CD8+ T lymphocytes without OVA peptide, untreated DCs:CD4+ or CD8+ T lymphocytes plus OVA peptide, and LPS-pretreated DCs:CD4+T lymphocytes plus OVA

peptide. OVA257-264 peptide was used to stimulate OT-I CD8+ T lymphocytes at 10 ng/mL, and OVA323-339 peptide was used to stimulate OT-II CD4+ T lymphocytes at 20 ng/mL. Cells were cultured during 48 hours at 37°C with 5% CO2. After, cells were collected and analyzed by flow cytometry (BD FACSCantoII) using following antibodies: anti-CD4-PerCP (clone RM4-5; BD Bioscience), anti-CD8-PerCP (clone 53-6.7; BD Bioscience), anti-CD25-PE (clone 7D4; BD Bioscience) and CD69-APC (clone H1.2F3; BD Bioscience).

Finally, all supernatants were collected and stored at -20C° until future use for cytokine measurement. For that, samples were thawed to perform cytokine quantification using OptEIATM ELISA kits (BD Pharmingen) for IFN-γ, IL-17, and IL-10, following the manufacturer instructions.

Serum Quantification of ALT, AST, and Aldosterone

Mice were euthanized according to ethics protocols. Blood was obtained through cardiac puncture. To obtain serum all samples were centrifuged at 14000 r.p.m. for 10 minutes (Jackson Laboratory guidelines). Alanine Transaminase (ALT) and Aspartate Transaminase (AST) were quantified with Kovalent Ltd. kit (Río de Janeiro, Brasil). Aldosterone was measured with ELISA kit Alpha Diagnostic International (San Antonio, TX, USA). All quantifications were performed following manufacturer guidelines.

Gene Expression by RT-PCR

Gene expression of proinflammatory cytokines (*Il-1β, Tnf-α, Ifn-γ, Il-18*), Mineralocorticoid receptor (*Mr*), and fibrosis markers (*α-Sma, Col1A, Mmp2, Tgf-β1,* and *Timp-1*) were measured in total RNA obtained from livers using TRIZOL reagent (Life Technologies Invitrogen, Carlsbad, CA) according to the manufacturer instructions. Then, 1 μg of total RNA was reverse-transcribed into cDNA using kitSuperScript VILO MasterMix (Thermo Fisher, USA). The cDNA template equivalent to 100 ng of total RNA was used to real-time PCR using Fast SYBR Green qPCR Master Mix plus specific gene primers (Thermo Fisher Scientific, NY, USA) in a StepOnePlus thermocycler (Applied Biosystems). Mouse 18S was amplified as an endogenous gene control. RT-PCR

conditions included an initial denaturation of the template at 95°C for 20 seconds minutes followed by 40 amplification cycles each consisting of denaturation at 95°C for 3 seconds followed by annealing and extension at 60°C for 60 seconds. Analysis were performed using 2 (-ΔΔCt) method. The primers used are listed in Supplementary Table 1.

References

1. Riquelme SA, Bueno SM, Kalergis AM. Carbon monoxide down-modulates Toll-like receptor 4/MD2 expression on innate immune cells and reduces endotoxic shock susceptibility. *Immunology* (2015) **144**:321–332. doi:10.1111/imm.12375
2. Guo W, Sun J, Jiang L, Duan L, Huo M, Chen N, Zhong W, Wassy L, Yang Z, Feng H. Imperatorin Attenuates LPS-Induced Inflammation by Suppressing NF-$κ$B and MAPKs Activation in RAW 264.7 Macrophages. *Inflammation* (2012) **35**:1764–1772. doi:10.1007/s10753-012-9495-9

Supplementary table 1. list of pcr primers for gene expression studies.

Gene	Primer sequence
α-Sma	F: GTC CCA GAC ATC AGG GAG TAA<br><br>R: TCG GAT ACT TCA GCG TCA GGA
Col1a1	F: GCT CCT CTT AGG GGC CAC T<br><br>R: CCA CGT CTC ACC ATT GGG G
Mmp2	F: CAA GTT CCC CGG CGA TGT C<br><br>R: TTC TGG TCA AGG TCA CCT GTC
Timp1	F: GCA ACT CGG ACC TGG TCA TAA<br><br>R: CGG CCC GTG ATG AGA AAC T
Il-1β	F: ACT GTT CCT GAA CTC AAC T<br><br>R: ATC TTT TGG GGT CCG TCA ACT
Tnf-α	F: CCC TCA CAC TCA GAT CAT CTT CT R: GCT ACG ACG TGG GCT ACA G
Il-18	F: GAC TCT TGC GTC AAC TTC AAG G<br><br>R: CAG GCT GTC TTT TGT CAA CGA
Ifn-γ	F: ATG AAC GCT ACA CAC TGC ATC R: CCA TCC TTT TGC CAG TTC CTC
Tgf-β1	F: CTC CCG TGG CTT CTA GTG C<br><br>R: GCC TTA GTT TGG ACA GGA TCT G
MR	F: GAAAGGCGCTGGAGTCAAGT<br><br>R: TGTTCGGAGTAGCACCGGAA
18s	F: TGA CGG AAG GGC ACC ACC AG<br><br>R: CAC CAC CAC CCA CGG AAT CG

Supplementary Results

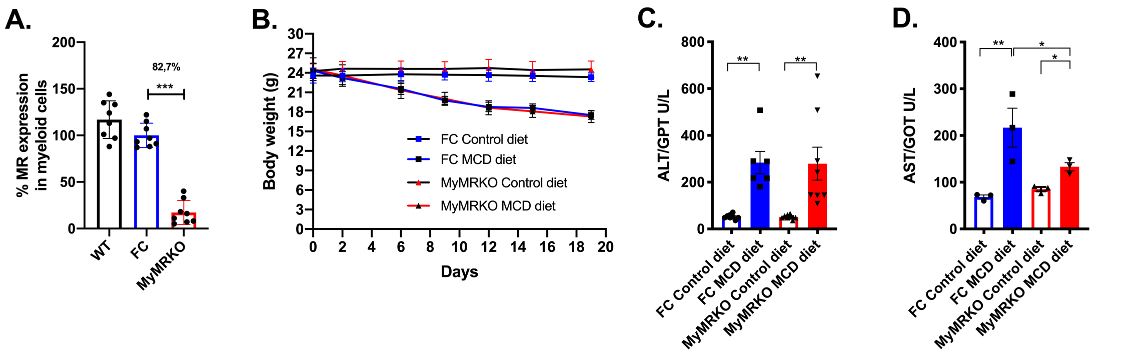

Supplementary Figure 1. MyMRKO and FC mice fed with MCD diet develop liver disease. A) Expression levels of MR in blood-isolated myeloid cells from WT, FC and MyMRKO mice respectively. B) Net bodyweight lost following MCD diet. Serum levels of hepatic function enzymes C) ALT/GPT, and D) AST/GOT. Both enzymes were significantly increased in both groups of MCD diet-fed mice. To quantify AST, it was necessary to pool plasma from 2 mice per group to achieve the required volume for quantification. For that reason, graph depicts n=4 pooled plasma samples for AST. ALT was measured individually in n=7-8 animals. Statistical analysis was performed with one-way ANOVA comparing all treatments, with Tukey post-test. All figures display mean +/- SEM. Statistically significant differences were considered according to *$p<0.05$ **$p<0.01$

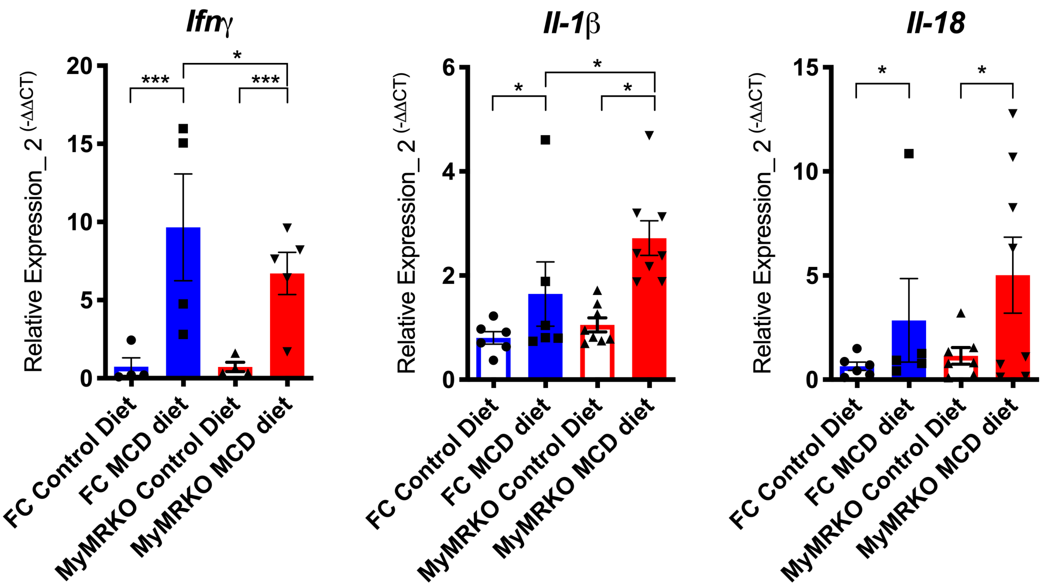

Supplementary Figure 2. FC mice fed with MCD display higher levels of T-associated cytokines and MyMRKO mice fed with MCD have higher levels of innate-associated cytokines. Markers of tissue inflammation such as Ifn-γ, Il-1β, and Il-18 were measured by RT- PCR in the liver at the endpoint. 18S was used as a housekeeping gene. Each gene expression quantification was performed in duplicate from an n= 7-8 animals per group. Statistical analysis was performed with one-way ANOVA comparing all treatments, with Tukey post-test. All figures display mean +/- SEM. Statistical differences were considered significant according to *p<0.05, ***p<0.001, ****p<0.0001.

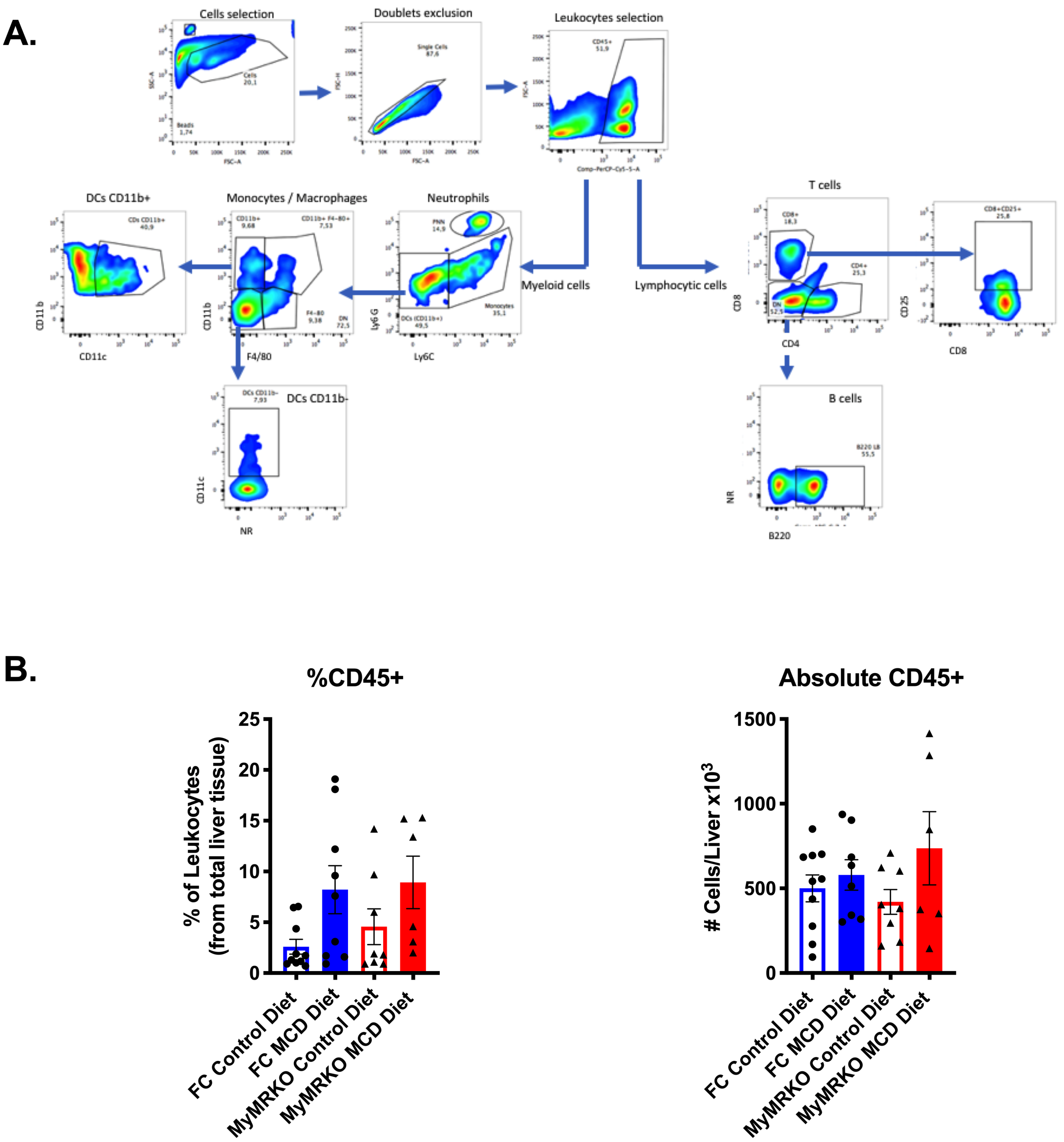

Supplementary Figure 3. Flow cytometry rationale to evaluate the leukocyte infiltration in livers. A) Gating strategy applied to analyze the cellular infiltrate in livers. B) Absolute number and percentage of leukocyte infiltration (CD45+ cells). Flow cytometry characterization was performed individually for each animal per group, achieving n= 7-8. Absolute counting was performed with Count BrightTM absolute counting Beads (described in Materials and Methods). Statistical analysis was performed with one-way ANOVA comparing all treatments, with Tukey post-test. All figures display mean +/- SEM. Differences were considered statistically significant according to *p<0.05 **p<0.01 ****p<0.0001.

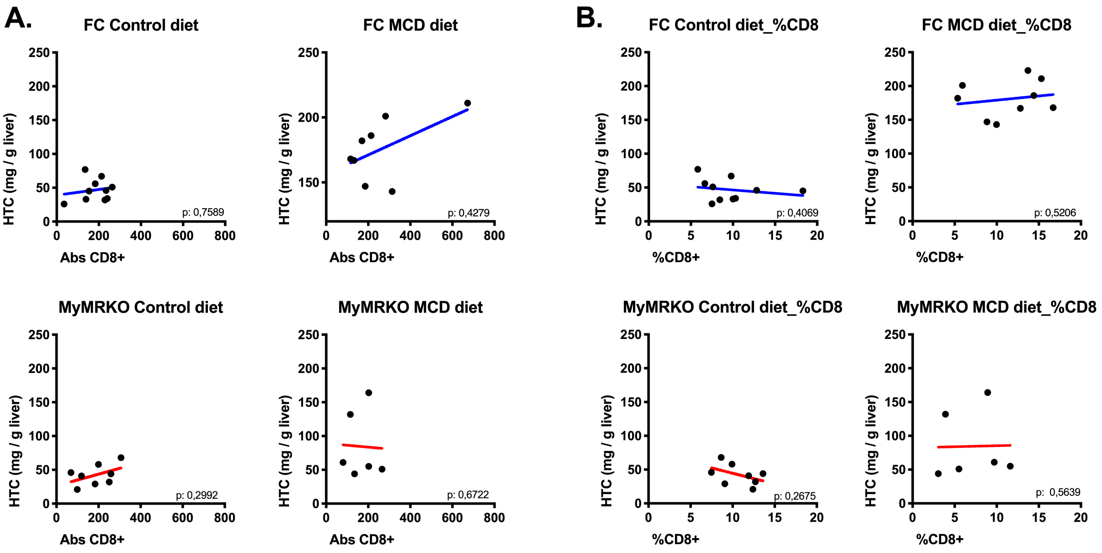

Supplementary Figure 4. Liver infiltration of CD8+ lymphocytes in FC mice fed with MCD diet tends to correlate with lipid accumulation in the liver. The correlation between the percentage (A) or absolute number (B) of CD8+ lymphocytes and hepatic triglycerides were measured through Spearman´s coefficient, considering an alpha = 0.05. Statistical differences were deemed to be significant according to *$p<0.05$.

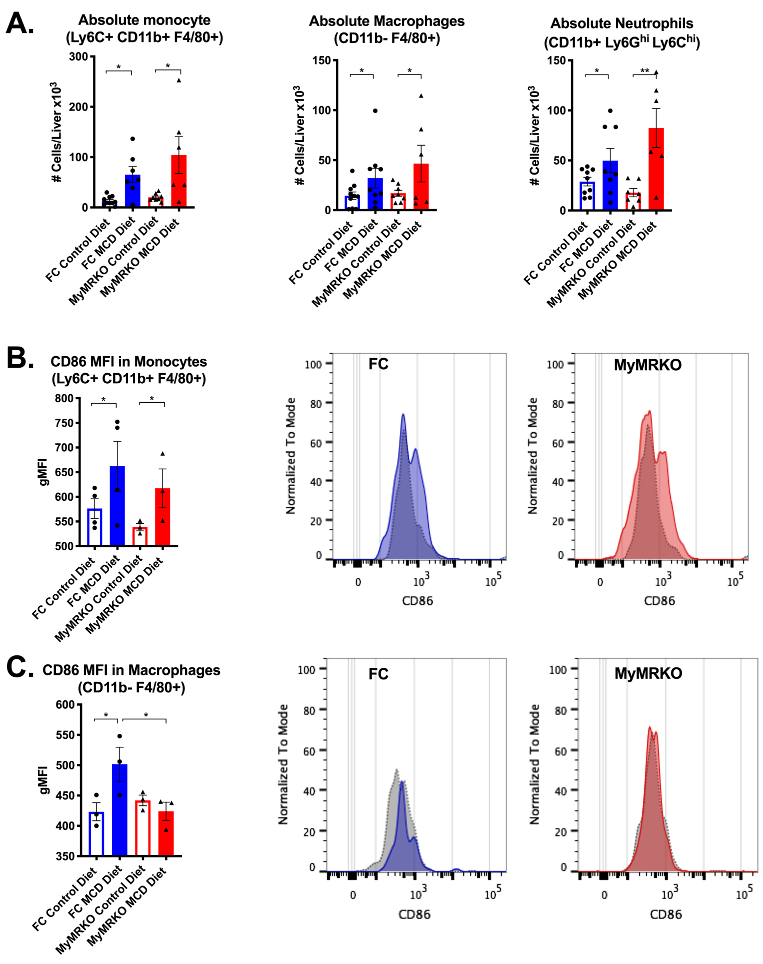

Supplementary Figure 5. Innate cells are significantly increased in MCD fed mice and tend to present an immunogenic phenotype. Flow cytometry was used to measure the absolute number of different subsets of myeloid cells (A), such as monocytes (left panel), macrophages (middle panel), and neutrophils (left panel). Additionally, surface expression of the costimulatory molecule CD86 was measured in antigen-presenting cells such as monocytes (B) or macrophages (C). For each cellular subset, a representative image of CD86 expression is shown in FC and MyMRKO fed with chow diet (grey dotted histogram) or MCD diet (colored histogram). Statistical analysis was performed with one-way ANOVA comparing all treatments, with Tukey post-test. All figures display mean +/- SEM. Statistical differences were considered significant according to *p<0.05, **p<0.01.

A.

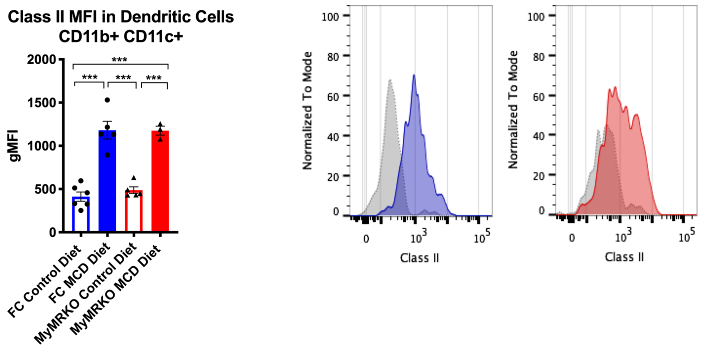

B.

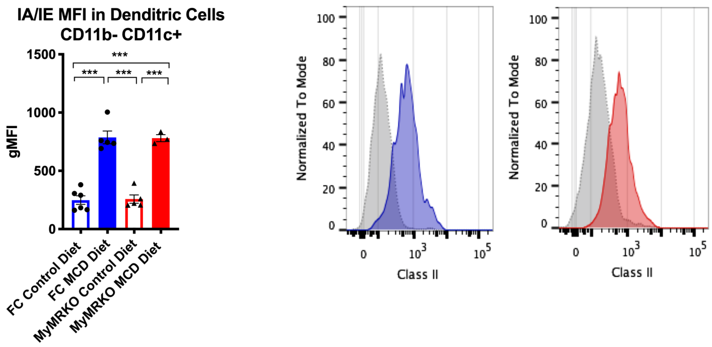

Supplementary Figure 6. DC subsets in liver of mice fed with MCD diet normally expressed IA/IE in inflamed animals. Flow cytometry was used to measure the intensity of IA/IE expression (gMFI) in two subsets of DCs: CD11b+CD11c+ (A) and CD11b-CD11c+ (B). For each cellular subset, a representative image of CD86 expression in FC and MyMRKO fed with chow diet (gray dotted histogram) or MCD diet (colored histogram) is shown. Statistical analysis was performed with one-way ANOVA comparing all treatments, with Tukey post-test. All figures display mean +/- SEM. Differences were considered statistically significant according to *$p<0.05$ **$p<0.01$ ****$p<0.0001$.

12 hours of DCs stimulation

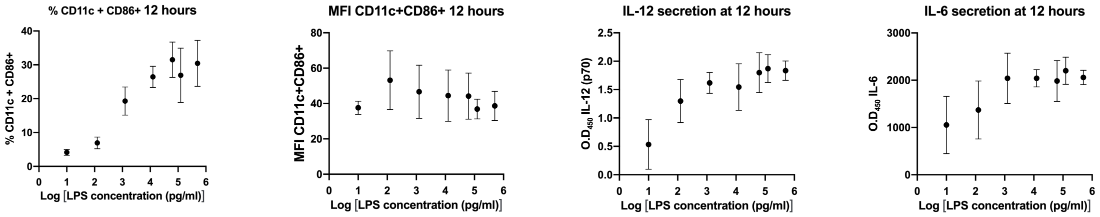

24 hours of DCs stimulation

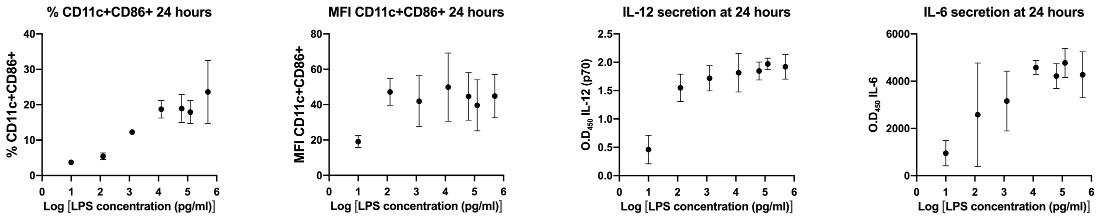

Supplementary Figure 7. LPS Dose-response curve. Bone marrow DCs cells were differentiated in in vitro with GM-CSF and then stimulated with different concentrations of LPS (0 ng/ml; 0,125 ng/ml; 1,25 ng/ml; 12,5 ng/ml; 62,5ng/ml; 125ng/ml and 500ng/ml) during 12 or 24 hours to define the minimum LPS concentration required to obtain a mature phenotype. This phenotype was measured through the surface maturation marker expression of CD86 by flow cytometry and cytokine secretion of IL-12 and IL-6 in supernatant by ELISA. LPS concentrations were plotted using a logaritmic scale and expressed in pg/ml.

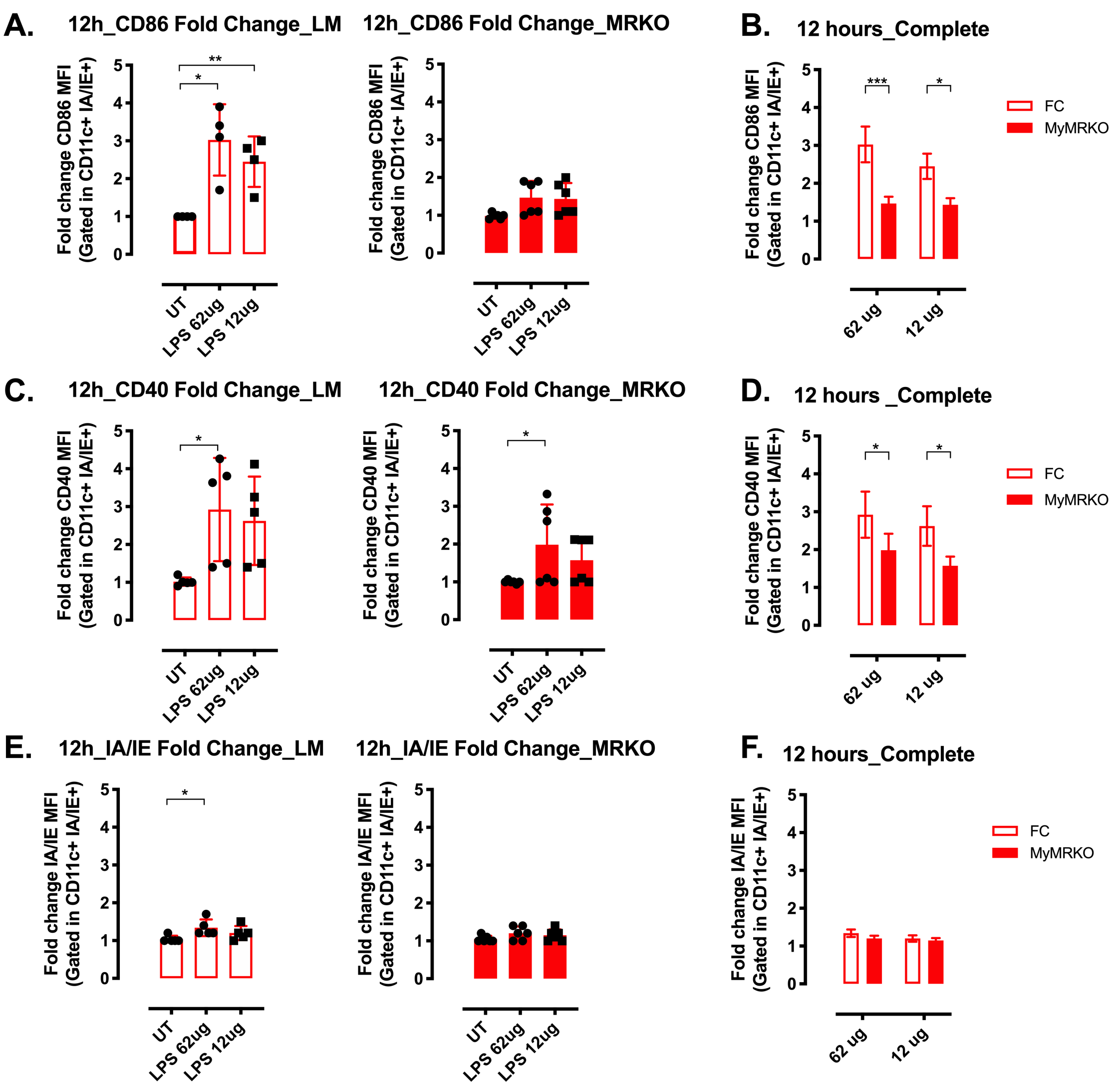

Supplementary Figure 8. Evaluation of surface maturation markers in DCs. DCs derived from MyMRKO mice showed a lower ratio of maturation in response to LPS challenge as compared to FC dendritic cells. DCs were derived from bone marrow FC, and MyMRKO. On the 5th day of DCs culture, it was pulsed with 62 µg/mL or 12 µg/mL of LPS for 12 hours. Followed the stimulation time, DCs were collected and stained with CD11c, IA/IE, CD86, and CD40 to measure by flow cytometry the expression of maturation marker. Mean fluorescent intensity (MFI) was calculated for CD86 (A), CD40 (C), and IA/IE (E), and results were expressed as fold change as compared untreated cells. Summarized results obtained for each maturation marker are shown in B, D, and F. Statistical analysis for A, C, and E were performed with one-way ANOVA comparing all treatments to untreated cells, with Dunnett post-test. Otherwise, interpretation of B, D, and F, were performed using a two-way ANOVA with Tukey post-test.

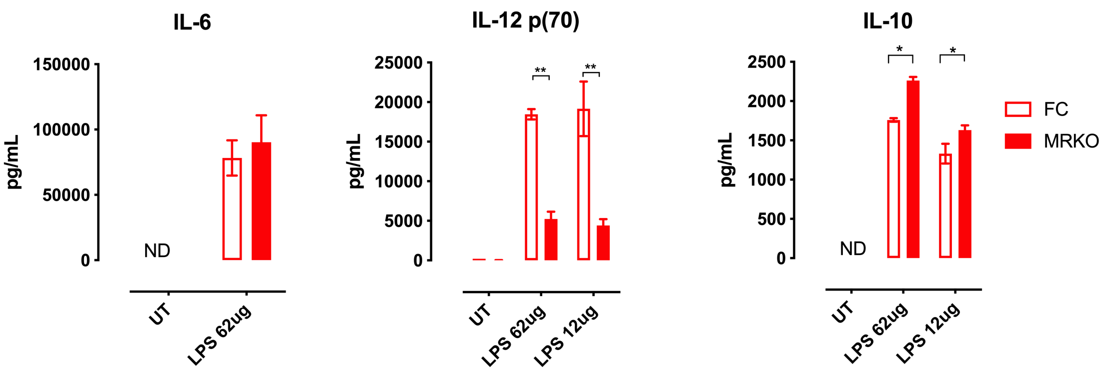

Supplementary Figure 9 Evaluation of Cytokine production in DCs derived from MyMRKO mice. MyMRKO DCs and mice displayed a tolerant response to the LPS challenge. DCs were derived from bone marrow FC, and MyMRKO. On the 5th day of DCs culture, it was pulsed with 62 μg/mL or 12 μg/mL of LPS for 12 hours. Followed the stimulation time, supernatants were collected to quantify by ELISA the levels of IL-6 (left panel), IL-12 p(70) (middle panel), and IL-10 (right panel). Statistical analyses were performed with two-way ANOVA, followed by Tukey post- test (n=6 replicates).

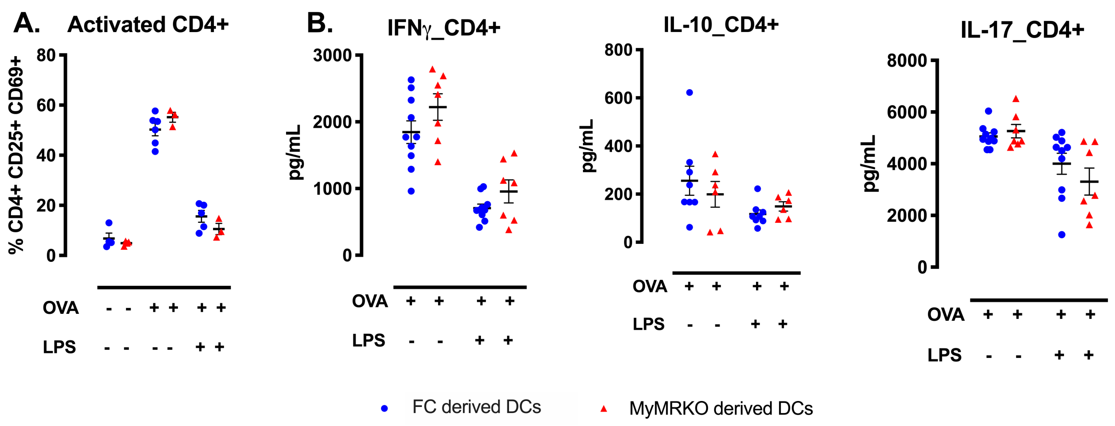

Supplementary Figure 10. Cocultures between OT-II lymphocytes and bone marrow derived DCs from MyMRKO or FC displayed similar results. Purified CD4+ OT-II lymphocytes were cocultured with DCs in a ratio of 1:1 for 48 hours. Three different conditions were tested: cocultures with DCs:CD4+T lymphocytes without OVA peptide, untreated DCs:CD4+T lymphocytes plus OVA peptide, and LPS-pretreated DCs:CD4+T lymphocytes plus OVA peptide. A) Expression of activation markers CD69 and CD25 in CD4+ T lymphocytes. B) Cytokine secretion for IFN-γ (left panel), IL-10 (middle panel), and IL-17 (right panel) in those supernatants by ELISA. Statistical analysis was performed with two-way ANOVA comparing all treatments, with Tukey post-test. All figures display mean +/- SEM. Differences were considered statistically significant according to *$p<0.05$.
